# Sexual behavior dysfunction and sickness behavior as an early warning indicators of imidacloprid exposure, associated with pituitary–adrenal axis dysregulation and DNA damage in Japanese quails

**DOI:** 10.3389/fvets.2026.1914494

**Published:** 2026-10-16

**Authors:** Ramadan D. El Shoukary, Doaa S. Abd El-Maguid, Asmaa H. Abd El Kareem, Aymen S. Abd El-Maguid, Gehad N. Seddik, Asmaa F. Khafaga, Nora Abdelbaeth

**Affiliations:** 1 Department of Animal Wealth Development, Faculty of Veterinary Medicine, New Valley University, New Valley, Egypt; 2 Department of Forensic Medicine and Toxicology, Faculty of Veterinary Medicine, New Valley University, New Valley, Egypt; 3 Department of Physiology, Faculty of Veterinary Medicine, New Valley University, New Valley, Egypt; 4 Department of Agricultural Economics, Higher Institute for Agricultural Cooperation and Extension, Assiut University, Assiut, Egypt; 5 Department of Animal Husbandry and Behaviors, Faculty of Veterinary Medicine, Sohag University, Sohag, Egypt; 6 Department of Pathology, King Faisal University, College of Veterinary Medicine, AL-Ahsa, Saudi Arabia

**Keywords:** comet assay, ecotoxicology, endocrine disruption, mating efficiency, neonicotinoids, pre-copulatory behavior, reproductive toxicity

## Abstract

**Introduction:**

Japanese quails (*Coturnix coturnix japonica*) serve as vital models in behavioral neuroendocrinology and ecotoxicology, particularly for understanding the neuroendocrine pathways regulating avian mating strategies. Subchronic exposure to neonicotinoid insecticides poses critical threats to avian reproduction and population dynamics.

**Methods:**

This study comprehensively evaluated the neuroendocrine, behavioral, morphometric, and genotoxic impacts of chronic oral imidacloprid (IMI) exposure in Japanese quail (*Coturnix coturnix japonica*) across developmental and adult stages (weeks 7–12 of age).

**Results:**

Imidacloprid administration significantly delayed pubertal onset and increased body weight at sexual maturity in both sexes (*p* ≤ 0.003). Morphometric analysis revealed profound somatic and gonadal hypotrophy, evidenced by persistent reductions in the brain-somatic index (BSI) and gonado-somatic index (GSI; testicular and ovarian masses) across development (*p* < 0.0001). Endocrine profiling demonstrated systemic disruption of the hypothalamic–pituitary–gonadal (HPG) axis, marked by significant suppression of circulating anterior pituitary gonadotropins (FSH and LH), testosterone, progesterone, and total estrogens (*p* < 0.0001), alongside biphasic 17β-estradiol deregulation. Behaviorally, exposed birds exhibited pronounced hypodipsia, increased stationary crouching, and progressive sickness behavior (*p* < 0.001). In males, IMI induced severe reproductive and behavioral deficits, characterized by marked reductions in cloacal gland area (304.66 vs. 332.90 mm), relative testis mass, and merocrine foam production (*p* < 0.001), accompanied by significant suppression of appetitive courtship displays (chasing, waltzing, wing flapping) and consummatory copulatory performance (mounting, cloacal contact movements, libido scores, and copulatory efficiency) in both rearing cages and standard testing arenas (*p* < 0.05 to *p* < 0.0001). In females, IMI significantly blunted proceptive squatting and holding-still compliance scores during mounting encounters without altering running escape frequency, alongside severe reductions in total weekly egg production, egg weight, hen-day egg production, and total egg mass (*p* < 0.001). Furthermore, the alkaline comet assay revealed marked hepatic and cerebral genomic DNA damage, with significant elevations in percentage DNA damage and tail moment (p < 0.05 to p < 0.001).

**Conclusion:**

Overall, these findings demonstrate that chronic imidacloprid exposure induces multi-level neuroendocrine disruption, systemic genotoxicity, and severe reproductive failure in avian models, highlighting significant ecotoxicological risks to non-target avian species.

## Graphical abstract

Integrated workflow and key findings.
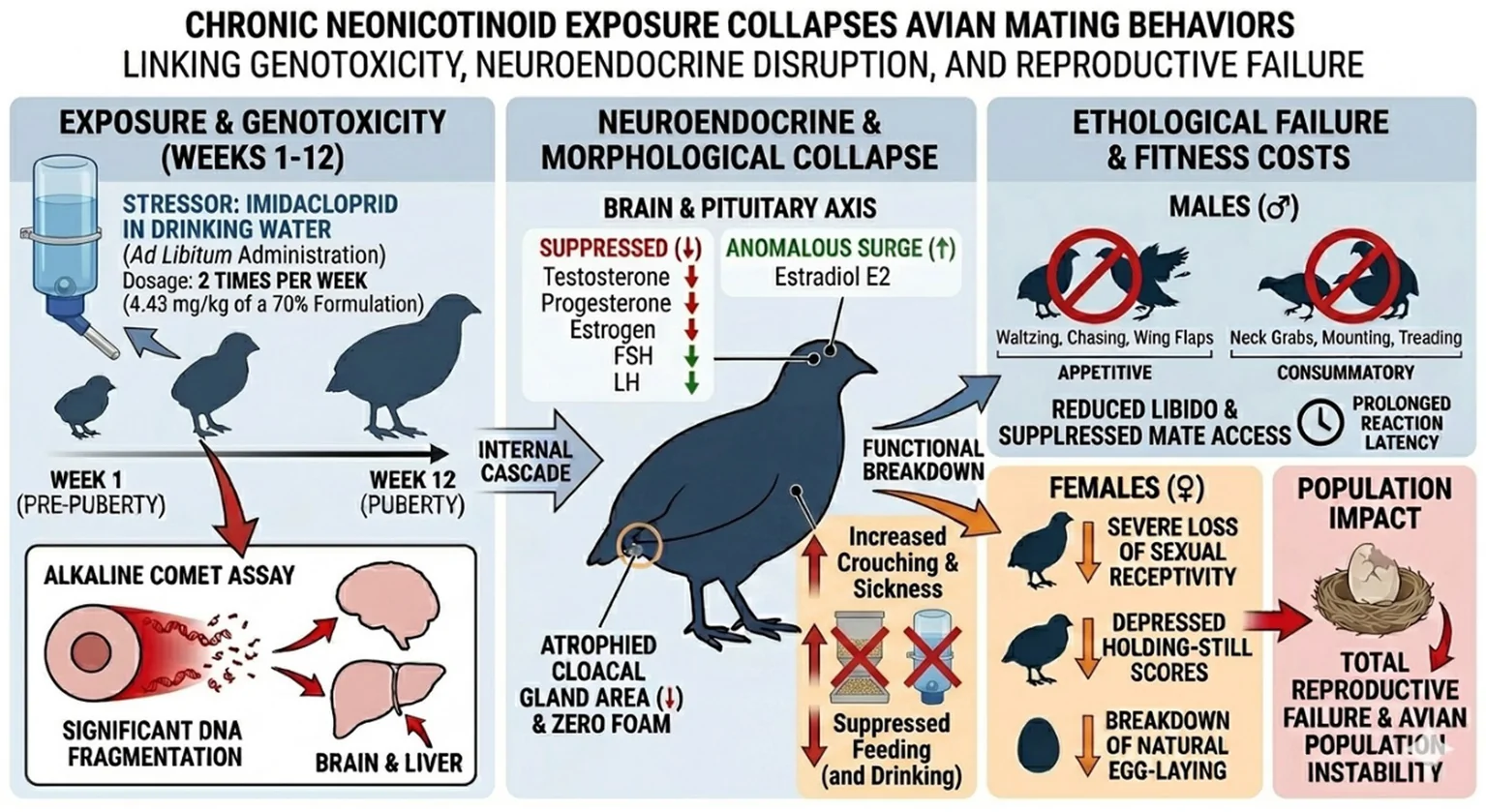

## 1 Introduction

Behavioral ecotoxicology is the study of behavioral responses used to determine the potential effects of toxicants and other stressors on individuals, populations, and communities (1). This field combines an understanding of how individuals adaptively respond to environmental stressors particularly chemical contaminants with the resulting impacts of these behavioral changes across various levels of ecological organization. Crucially, variation in behavioral traits measured in laboratory settings can predict the fitness of individuals in the wild (2).

Endocrine-disrupting pesticides (EDPs), such as imidacloprid (IMI), are widely used in agriculture and veterinary medicine to enhance productivity by combating devastating insect pests (3). However, EDPs interfere with the synthesis, secretion, or action of endogenous hormones, thereby disrupting endocrine function in target organisms (4). Beyond its intended agricultural use, Imidacloprid (IMI) has become one of the most widely used neonicotinoids and frequently detected environmental contaminants in agricultural soils, surface waters, and non-target wildlife habitats globally (5). It induces oxidative stress and acts as an environmental stressor (6) that inadvertently affects non-target organisms, including mammals.

Humans are primarily exposed to IMI through the inhalation of household dust, the oral ingestion of contaminated food and water (7), or direct contact with treated companion animals (8).

Infertility is a major global health concern, affecting nearly 10–15% of reproductive-aged couples and exerting significant psychosocial and economic burdens (9). Previous studies have concluded that IMI can exert adverse impacts on reproductive functions (10), leading to systemic reproductive toxicity (11) and infertility in various animal and bird species (79). Furthermore, IMI disrupts the hypothalamic–pituitary axis, which impairs the endocrine regulation of the thyroid and testes (12). This disruption ultimately compromises the regulation of male reproductive behaviors and secondary sexual characteristics by decreasing the levels of essential sex hormones like testosterone (6) and disrupting metabolic homeostasis and steroidogenesis via the inhibition of cytochrome P450 enzyme activities (3).

Quails serve as excellent bio-indicators of environmental contamination, including imidacloprid (IMI) exposure (13), and offer distinct economic advantages such as low maintenance costs, strong disease immunity, and high egg production rates (14). Consequently, the genital tracts of developing male and female quails provide an ideal model for investigating pubertal morphological changes induced by IMI (6, 78, 79).

Extensive research has documented the diverse effects of IMI on avian reproductive, productive, and fertility parameters across both sexes. In female birds, IMI significantly reduces the fertility index, alters circulating reproductive hormones, causes ovarian and oviductal structural damage (15), disrupts ovarian folliculogenesis, and decreases overall ovarian follicle numbers (6, 8). These structural anomalies are mediated by severe endocrine disruptions, including diminished concentrations of progesterone and estrogen (16), decreased luteinizing hormone (LH) levels, and paradoxical elevations in follicle-stimulating hormone (FSH) (4) and estradiol (*E*2) levels (6) collectively resulting in adverse reproductive health outcomes such as reduced clutch sizes, impaired egg viability, lower hatchability (17), and decreased egg size (18). Parallelly, male exposure adversely targets nearly every element of the reproductive system, causing severe degeneration and necrosis of both germ cells and Leydig cells, alongside widespread atrophy of the seminiferous tubules (19), as well as general testicular and epididymal damage (4, 10).

Physiologically, these manifestations present as a significantly lower testicular weight (10), decreased testosterone concentrations (6), and downregulated androgen receptors (20).

Ultimately, this cascade severely impairs sperm quality by disrupting spermatogenesis, altering sperm morphology, increasing mortality, and reducing overall sperm count and motility, thereby drastically compromising overall fertility (4).

Behavioral neuroendocrinology explains the relationship between hormones and behavior (21), and the Japanese quail (*Coturnix japonica*) serves as a highly effective avian model for studying both behavioral neuroendocrinology and ecotoxicology, particularly regarding the neuroendocrine control of reproductive and social behaviors (22). This species acts as a foundational model system for investigating the steroid-catecholamine interactions that mediate both the appetitive and consummatory aspects of male sexual behavior (23), which are recognized as the most sensitive endpoints when evaluating toxicity in male Japanese quail (24). Mature male Japanese quails display distinct courtship and vigorous mating behaviors under standardized laboratory conditions, making them excellent subjects for analyzing normal reproductive dynamics and deviations from them, which ultimately provides valuable insights for understanding human sexuality and behavior (7).

Despite a substantial body of research documenting the reproductive toxicity and reduced fertility associated with imidacloprid (IMI), the specific effects of oral IMI administration during the pre-pubertal or pubertal periods on general sickness behaviors or male sexual behaviors remain unassessed. These unstudied endpoints include courtship behaviors (such as struts, waltzes, and wing flapping), copulatory actions (including neck grabs, mount attempts, mounts, and cloacal contact movements), mating efficiency, sexual power, sexual experience, sexual development parameters, and female-specific reproductive behaviors like receptivity and egg-laying.

Consequently, the current study utilizes quail sexual behavior as a highly sensitive endpoint for detecting neuroendocrine-disrupting contaminants, given that these behaviors consist of well-defined, easily quantifiable sequences under laboratory conditions. This research focuses primarily on tracking alterations in behavioral signs in Japanese quail and correlating them with hormonal, pathological, or neurological changes induced by IMI. Because behavioral expressions are direct manifestations of underlying neurological functions, behavioral analysis is crucial for evaluating neural integrity (25). Furthermore, behavioral responses serve as vital “early warning signals” because they are highly flexible, rapid traits that frequently manifest at significantly lower chemical exposure levels than traditional toxicological endpoints (26).

Behavior represents the final, integrated phenotypic outcome of complex biochemical and physiological pathways (27). Therefore, evaluating behavior offers a non-invasive, cost-effective, and highly sensitive proxy for health that can detect toxic insults well before structural changes register in other phenotypes or the genome, allowing environmental and physiological disruptions to be more accurately tracked (26).

We hypothesized that chronic, juvenile and pubertal exposure to IMI would alter specific courtship and copulatory sequences before gross morphological changes occurred.

## 2 Materials and methods

### 2.1 Chemicals insecticide used

Commercial-grade imidacloprid (IMI; trade name: Joun 70% WG) was obtained from Aspire Agro Chemicals Company. The experimental treatment dose was calculated and established at an amount equivalent to 1/10 th of the determined (LD_50_) following the sub-chronic dosing protocol outlined by Emam et al. (28).

### 2.2 Ethical approval and site of experiment

The study was conducted at the animal housing facility of the Faculty of Veterinary Medicine, New Valley University, Egypt. All experimental protocols and animal handling procedures were reviewed and approved prior to study initiation by the Institutional Animal Care and Use Committee of New Valley University (Approval No. NVIRE-SIC/IACUC/3/6–2026/02/1), in strict compliance with national guidelines for the care and use of laboratory animals.

### 2.3 Birds and management

A total of 320 one-day-old quail chicks, with an initial body weight of 8 ± 0.02 g, were obtained from the Faculty of Agriculture at New Valley University.

Upon arrival, the chicks were housed in a galvanized, six-tier battery cage system consisting of three cages per tier, with each cage measuring 50 × 50 × 30 cm (length × width× height).

To accommodate the chicks during the first two weeks, a plastic mesh was secured to the sides and floor of each cage and the floor was bedded with a 5 cm layer of sawdust.

Flock management was conducted in accordance with the protocols described by Elbaz et al. (29). Specifically, the ambient temperature was initially set at 37 °C and systematically decreased by 3 °C per week. The photoperiod consisted of a 23-h light and 1-h dark cycle. Throughout the 42-day rearing period, the quails were provided ad libitum access to water and a standard quail starter diet formulated to contain 24% crude protein and 12.45 MJ/kg of metabolizable energy, satisfying the nutritional requirements outlined by the National Research Council (NRC, 1994). Following the rearing phase, the birds were sexed at a 1:1 ratio, and management protocols were adjusted to accommodate production status according to Hafez et al. (30). During this phase, the experimental rooms utilized natural light to maintain the birds in reproductive condition (Supplementary Figure S1).

### 2.4 Experimental design

Brooding Phase (Weeks 0–6): Day-old quail chicks were randomly assigned to two treatment groups, with eight replicates per group (*n* = 20 chicks per replicate). The control group received standard drinking water, while the treatment group received an oral administration of imidacloprid (1/10 LD_50_; 4.43 mg/kg of a 70% formulation) dissolved in water. Dosing was conducted twice weekly (Sundays and Thursdays) from hatch through week 12.

Production Phase (Weeks 7–12): At 6 weeks of age (onset of sexual maturity), birds were redistributed to establish a 1:1 male-to-female sex ratio. To maintain group size while accommodating this post-maturation rebalancing, the design was adjusted to six replicates per group (*n* = 20 birds per replicate). Using a stratified randomization scheme within treatment tiers, birds were reassigned into adult sub-cages to ensure equal body-weight distributions across sub-replicates. To prevent territorial aggression, facilitate sex-specific testing, and avoid cross-contamination, birds were transferred strictly within their original imidacloprid exposure tiers. Data collection for this secondary phase occurred between days 49 and 84 (weeks 7–12), following complete physiological maturation.

Starting from week 7 through week 12, destructive sampling was conducted weekly. To maintain independence and avoid pseudoreplication, exactly one male and one female were randomly sampled from 5 randomly selected replicate from the 6 replicate cages per treatment group each week (*n* = 5 birds/sex/treatment/week). Since each sampled bird originated from a distinct, independent replicate cage and no cage was re-sampled for the same biological assay across subsequent weeks, individual birds served as the observational units for the weekly cross-sectional analyses, while the cage served as the true experimental unit for treatment administration (Supplementary Figure S2).

### 2.5 Assessment of sexual maturity

The quails were monitored daily between 36 and 45 days of age to determine the onset of sexual maturity. Individual males were deemed reproductively mature on the first day foam production was observed at the cloacal gland, while females were considered mature upon the onset of egg laying, Upon reaching sexual maturity, the age (days) was recorded, and individual body weight (g) was measured using a digital scale accurate to the nearest 0.1 gram. Additionally, the overall onset of maturity for the experimental groups was identified when the first hen in the cohort produced an egg, in accordance with Reddish et al. (31).

### 2.6 Behavior observation and evaluation tests

#### 2.6.1 In-cage Behavioral observations

Behavioral patterns were monitored using a continuous visual scan-sampling technique adapted from Mattsson and Brunström (32). Observations were conducted on the day following imidacloprid administration across six consecutive weeks (weeks 7–12 of age). Quails were observed for 4 h per day, 2 days per week, with each replicate monitored for 10 min during either the morning or afternoon period. To capture representative daily activity, each observation day was divided into morning (06:00–12:00 h) and afternoon (12:00–18:00 h) intervals. Monitoring was performed for 2 h during each interval using a rotational schedule modified from Hafez et al. (30) and Elsanhoury et al. (33).

Specifically, on the first observation day of each week, monitoring occurred from 06:00 to 08:00 h and 12:00 to 14:00 h, whereas on the second day, observations took place from 08:00 to 10:00 h and 14:00 to 16:00 h. Hourly observation blocks were subdivided into 5-min scan-sampling intermissions to record all behaviors within a replicate, immediately followed by a subsequent 5-min monitoring interval (34). The mean frequency for each behavior was calculated across six replicates, and the relative behavioral proportion was calculated using Equation: Behavioral (Proportion) = Frequency of Specific Behavior/Total Behavioral Frequencies.

#### 2.6.2 Observer blinding and video recording

To eliminate observer bias, all behavioral trials were conducted under strict double-blind conditions. Treatment groups were assigned randomized alphanumeric codes by an independent researcher not involved in behavioral scoring, and codes were unblinded only after statistical analyses were finalized. Arena testing sessions were continuously recorded using a high-definition digital camera positioned 1.5 m directly in front of an unobstructed glass viewing panel. High-speed digital video recordings were archived and subsequently analyzed frame-by-frame using digital event-logging software to quantify short-duration kinetic events (e.g., neck grabs and cloacal contact movements).

#### 2.6.3 Inter-observer reliability

Behavioral quantification was performed independently by two trained observers. Prior to formal trial scoring, inter-observer reliability was validated using a subset of 10 pilot videos. Inter-rater agreement was evaluated using Cohen’s kappa (κ) for discrete behavioral frequencies (e.g., waltzing, mount attempts, receptivity postures) and the two-way mixed Intraclass Correlation Coefficient (ICC) for continuous temporal parameters (e.g., latency, reaction time). Strong inter-observer consistency was established prior to formal data collection (kappa = 0.88; ICC = 0.92). In cases where scoring discrepancies exceeded 5% during formal trial analysis, recordings were jointly reviewed by both observers to reach a consensus.

##### 2.6.3.1 General behaviors (sickness behavior)

During the observation sessions, specific behavioral patterns were quantified based on the ethogram criteria adapted from Gormally et al. (35) the complete ethogram and behavioral definitions are detailed in Supplementary Table S1.

##### 2.6.3.2 Evaluation of sexual and copulatory behaviors

Male and female sexual behaviors were quantified during designated observation sessions in accordance with established avian ethological frameworks (30, 36). Male sexual behavior was categorized into appetitive (courtship/pre-copulatory) and consummatory (copulatory performance) phases to evaluate both central nervous system-regulated sexual motivation and neuromuscular execution (7). Female responses to male copulatory attempts were classified as either receptive or non-receptive. The complete echogram and detailed behavioral definitions are provided in Supplementary Table S2.

###### 2.6.3.2.1 Behavioral evaluations in an external testing arena (outside rearing cage)

Sexual behavior tests were conducted weekly when the quails reached 49, 56, 63, 70, 77, and 84 days of age. These evaluation points corresponded precisely to the 14th, 16th, 18th, 20th, 22nd, and 24th cumulative oral doses of imidacloprid, respectively. For each weekly testing session, spanning weeks 7 to 12 of age, 5 males and 5 females were randomly selected from each treatment group.

All behavioral trials were conducted in a strictly controlled testing room beginning at 11:00 h. The testing apparatus consisted of a glass-walled observation arena measuring (60 × 30 × 36 cm), (length × width × height). To eliminate external visual distractions and focus the birds’ orientation, three of the glass walls were completely blinded with opaque dark sheets, leaving only the front panel unobstructed for observation. The floor of the arena was bedded with clean grass, and the top was secured with wire mesh webbing.

Following a modified protocol adapted from Suzuki and Tsukahara (36), a single female quail was initially introduced into the arena and allowed an acclimation period of 5 min. Subsequently, a test male from the corresponding treatment group was introduced into the arena, and behavioral interactions were recorded for an additional 5 min. Behavioral tracking was performed via direct focal sampling to quantify the specified parameters. Immediately following the conclusion of each behavioral session, the tested birds were humanely slaughtered for subsequent physiological sampling.

#### 2.6.4 Sexual interaction, reaction time libido score and sexual performance (initial 2-min screening window)

Male sexual behavior, temporal interaction parameters, libido, and sexual performance incidence were evaluated during an initial 2-min screening window using a digital stopwatch (Supplementary Figure S3).

Recorded temporal metrics included latency to the first sexual interaction, reaction time, sexual interaction time, mount attempts, and cloacal contact movement. Specific behavioral definitions, scoring systems, and ethological criteria for each parameter are detailed in Supplementary Table S3.

#### 2.6.5 Male sexual behavior (full 5-min behavioral trial)

Only males that exhibited a minimum of one mount attempt and/or cloacal contact movement within the initial 2-min screening window were classified as sexually active and permitted to continue in the full 5-min behavioral trial. Throughout this entire 5-min tracking period, the absolute frequencies of discrete male sexual behaviors specifically neck grabs, mount attempts, mounts, and cloacal contact movements directed toward the female were continuously quantified using a direct focal sampling technique, following Suzuki and Tsukahara (36). For subsequent statistical analyses, the individual frequencies of each behavioral pattern were averaged across the five tested males per group to establish mean replicate values (36).

#### 2.6.6 Quantification of female sexual receptivity and postural behaviors

Female sexual receptivity, postural responses, and physical compliance were quantified from 5-min video recordings using three distinct scoring metrics. For all parameters, data were expressed as the mean value calculated across the five tested females per treatment group.

Baseline receptivity frequency was determined by recording male-initiated neck grabs and mount attempts alongside corresponding female behavioral responses (37), categorized as either receptive (squatting/crouching beneath the male) or non-receptive (active avoidance/running). Physical compliance and immediate reactivity were evaluated using two validated semi-quantitative scoring systems: a 4-point immobility scale (Scores 0–3) assessing the proportion of time the female remained stationary (38) and an initial receptivity scale (Scores 0–3) evaluating immediate postural responses upon male introduction (39). Detailed ethological criteria, ordinal scoring scales, and classification frameworks are summarized in Supplementary Table S4.

#### 2.6.7 Determination of copulatory efficiency and sexual experience indices

##### 2.6.7.1 Copulatory efficiency

Copulatory efficiency was calculated according to Marin and Satterlee (38) to measure the success rate of a copulatory sequence once it has been initiated: Copulatory efficiency = Total number of cloacal contact/Total number of neck grabs.

##### 2.6.7.2 Sexual experience index

The sexual experience index was calculated according to Can et al. (40) to quantify the average temporal efficiency required for a male to successfully achieve cloacal contact during behavioral interactions with the female:


$$\begin{array}{l} \text{Sexual experience index}=\text{Total number of cloacal contact}/\\ \text{sexual interaction time}\;(\text{second}) \end{array}$$

#### 2.6.8 Classification and success rates of mating behaviors

Complete Mating In this behavior the male quail grasps the back of female’s head and mounting attempts, with vent positioning or ejaculation while, Incomplete Mating In this behavior the male quail grasps the back of female’s head and mounting attempts without vent positioning or ejaculation (33).

.Copulatory outcomes were classified into two distinct behavioral categories based on the completion of the mating sequence, following the criteria of Elsanhoury et al. (33):

- Complete Mating: Defined as a copulatory sequence in which the male successfully grasps the feathers at the back of the female’s head, executes a mount attempt, and achieves proper vent positioning (cloacal alignment) resulting in ejaculation.
- Incomplete Mating: Defined as an interrupted or unsuccessful copulatory sequence in which the male grasps the back of the female’s head and attempts to mount, but fails to achieve vent positioning or successful ejaculation.

To evaluate group-level reproductive efficiency, the proportions of successful and unsuccessful mating behaviors were calculated as cohort percentages (*n* = 5 tested males per treatment group) using the following formulas: successful mating behavior % = number of male done complete mating/5 males, and unsuccessful mating behavior % = number of male do not do complete mating/5 males.

### 2.7 Estimation of productive or reproductive parameters

#### 2.7.1 Male fertility parameters

Five males at each week, which used in the previous sexual test, were used to measure cloacal gland area (CAREA mm^2^) and cloacal gland foam production (CFP) weight in gram according to as, Cloacal or (proctodeal) gland area (CAREA mm2) were measured to nearest 0.01 mm by using the digital vernier caliber as the cloacal gland area was used as an index of gland size. As the cloacal gland areas (the longest length× the greatest width) as CAREA mm^2^ = (AG = LW × VDH), as (LW) mean the lateral width and (VDH) mean ventral-dorsal height of the cloacal gland which measured by using a digital caliper (41).

Quantitative measurements (weights) of the cloacal gland foam production (CFP) was collected individually by delicate, manual squeezing at the lateral wall of the foam gland with the thumb of the left hand and the forefinger of the right hand then the produced foam was collected on aluminum paper, which was put rapidly in airtight glass bottles to prevent evaporation of the foam and then weighed by using an electronic analytical balance in gram (41).

Five males at each week, which used in the previous sexual test, were used to measure Testosterone hormone levels were measured by Enzyme-Linked Immunosorbent Assay (ELISA) kits according to (30) according to the manufacturer’s instructions (MYM Laboratory and Medical Supply, Inc., San Diego, USA). The intra-assay coefficient of variation is 7.4 while, the inter-assay coefficient of variation is 5.2 with a minimum detectable concentration is 0.05 ng/ mL.

#### 2.7.2 Female fertility parameters

##### 2.7.2.1 Productive performance indices

The total number of eggs laid within each replicate was recorded daily, and individual egg weight was determined using an electronic precision balance to the nearest 0.1 gram. At the end of each weekly interval, the hen-day egg production rate (HDEP) and daily egg mass {g/hen/day} were calculated according to the formulas described by Hafez et al. (30)


$$\text{HDEP}\%=(\begin{array}{l} \text{Total number of}\;\mathrm{egg}\;\text{laid during the period}/\\ \text{Total number of live}\;\mathrm{hen} \end{array})\;\times \;100$$

##### 2.7.2.2 Female reproductive endocrine profiles

Blood samples were collected weekly from the five females per treatment group previously evaluated for behavioral testing to quantify circulating reproductive hormone profiles. Serum concentrations of follicle-stimulating hormone (FSH), luteinizing hormone (LH), progesterone, 17β-estradiol (E) and total estrogens were determined using commercial Enzyme-Linked Immunosorbent Assay (ELISA) kits. Analytical procedures for gonadotropins (LH and FSH) and prolactin strictly followed the manufacturers’ operational manuals and validated ethological protocols (42).

Serum 17β-estradiol (E) was quantified using a highly specific monoclonal antibody assay. In contrast, total estrogen concentrations were determined using a broad-spectrum polyclonal antiserum designed to cross-react with and capture multiple circulating estrogenic metabolites (E_1_, E_2_, and E_3_).

### 2.8 Ethical slaughter, blood collection, and sample processing

At the weekly conclusion of the experimental phases (weeks 7 to 12 of age), the five males and five females previously selected for behavioral trials were weighed and humanely slaughtered following halal (Islamic) slaughtering methods designed to minimize pain and distress. The procedure involved a rapid, continuous incision across the jugular veins and carotid arteries using a sharp blade, and the carcasses were suspended vertically to ensure complete exsanguination (43).

Whole blood was harvested immediately during exsanguination and divided into two distinct collection systems: non-additive tubes to obtain serum and tubes containing ethylenediaminetetraacetic acid (EDTA) acting as an anticoagulant to isolate plasma. Collected samples were immediately transferred to a cold storage environment maintained at 4 °C.

To separate the liquid fractions, all blood collection tubes were centrifuged at 3000 rpm for 15 min at 4 °C, The separated serum and plasma supernatants were immediately aliquoted into sterile Eppendorf tubes and transferred to a ultra-low temperature freezer maintained at −20 °C for storage until subsequent endocrine profiling. These samples were later utilized to quantify circulating levels of testosterone, luteinizing hormone (LH), follicle-stimulating hormone (FSH), progesterone, and total estrogens, and estradiol.

### 2.9 Carcass processing and somatic organ indices

Following complete exsanguination, the quail carcasses were immediately defeathered and manually eviscerated. Individual final body weights were recorded, and the brain, testes, ovary, and liver were harvested. Each organ was cleared of any adhering fat or connective tissue and weighed immediately using an electronic analytical balance to the nearest milligram.

To evaluate reproductive and physiological status relative to systemic mass, the gonadosomatic index (GSI%) and the brainsomatic index (BSI%) were determined according to the protocols of Hafez et al. (30). The relative weights of the organs were calculated using the following mathematical equations:

#### 2.9.1 Gonado-somatic index (GSI)

The GSI (representing the relative weight of the reproductive gonads) was calculated for males and females, respectively, as


$$\mathrm{GSI}\%=\{\begin{array}{l} \text{Testis or ovary weight}\;(g)/\\ \mathrm{pre}-\text{slaughter body weight}\;(g) \end{array}\}\times 100$$

#### 2.9.2 Brain-somatic index (BSI)

The BSI (representing the relative weight of the brain) was calculated as:


BSI%={Brain weight(g)/pre−slaughter body weight(g)}×100

### 2.10 Alkaline comet assay (single-cell gel electrophoresis)

To quantify single-strand DNA breaks and oxidative DNA damage, an alkaline comet assay was performed on the harvested brain and liver specimens following the protocols of (44) At the conclusion of the 12-week exposure period, fresh brain and liver tissue specimens were isolated and preserved in polypropylene vials containing a mixture of 1 mL normal saline + 5 microliter dimethyl sulfoxide. The tissue storage vials were maintained at −20 °C until subsequent cell isolation and processing.

#### 2.10.1 Slide preparation

Frosted slides were layered twice with 100 μL 1% GP-42 normal agarose (Nacalai Tesqe, Inc. Kyoto, Japan). Seventy-five microlitre (μL) of nuclear suspension was mixed with equal amount of 2% low melting (LGT) agarose (Nacalai Tesqe, Inc. Kyoto, Japan) at 45 °C and the mixture was layered on the frosted slide using a cover slide. Then, 100 μL of agarose GP42 was layered on the surface and covered with another slide and allowed to gel.

#### 2.10.2 Lysing

The slides were placed immediately into a chilled lysing solution (2.5 M NaCl, 100 mM Na4EDTA, 10 mM Trizma, 0.1% Sodium lauryl sulfate (SDS), 10% dimethyl sulfoxide, and triton X-100) and kept at 4 °C in the dark for 60 min.

#### 2.10.3 Unwinding and electrophoresis

The slides were arranged on a horizontal gel electrophoresis platform (Cleaver Scientific Ltd., U. K.) and covered with chilled alkaline solution (300 mM NaOH and 1 mM Na2 EDTA, pH 13) in the dark for 10 min, electrophoresis was conducted at 25 V and approximately 300 mA for 15 min. Then the slides rinsed with 400 mM Tris buffer (Wako Pure Chemical Industries, Ltd. Japan) pH 7.5 for 7 min. to neutralize the excess alkali. The neutralized slides were kept in ethanol for 5 min. Then allowed to dry at room temperature, then stained with 50 μL (20 μg/mL) ethidium bromide (Wako Pure Chemical Industries, Ltd.).

#### 2.10.4 Examination of the slides

The nuclei on the slides were examined at 200-fold magnification using the green filter of a fluorescence microscope (Olympus BX-43, Japan). The image of the nuclei was captured using digital camera. At least 50 cells per slide were analyzed using Comet Assay Software Project (CASP) to measure the diameter of the head, % of DNA in the tail and the length of tail of the comet to obtain DNA migration. Tail moment (TM) and Olive tail moment (OTM) were used as accurate indicators of DNA damage;


$$\text{Tail Moment}\;(\mathrm{TM})=\frac{\text{Tail Length}\times \%\text{Tail}\;\mathrm{DNA}}{100}$$

$$\text{Olive Tail Moment}\;(\mathrm{OTM})=\frac{(\begin{array}{l} \text{Tail Mean}-\\ \text{Head Mean} \end{array})\times \%\text{Tail}\;\mathrm{DNA}}{100}$$

### 2.11 Statistical analysis

An *a priori* sample size calculation was conducted to determine the required number of birds per experimental group using G*Power software (version 3.1.9.7). Based on pilot observations and previous avian toxicological studies evaluating organo-somatic indices, behavioral frequencies, and DNA damage parameters, a minimum effect size (f or d) ranging from 1.2 to 1.5 was anticipated. Setting the statistical power (1 - β) to 0.80 and the significance level (α) to 0.05, a sample size of *n* = 5 birds per sex per group (5 males and 5 females per treatment group) was determined to be sufficient to reliably detect statistically significant main effects and interactions.

To ensure statistical validity and prevent pseudoreplication, the cage was designated as the primary experimental unit across all analyses. From weeks 7 through 12, destructive sampling was conducted at weekly intervals. Five cages per treatment group were randomly selected each week, and one bird of each sex was sampled per cage (*n* = 5birds/sex/treatment/week). Because no cage was re-sampled for the same assay across subsequent weeks, individual birds served as the observational units for cross-sectional evaluations, while the cage represented the true experimental unit for treatment administration.

Prior to inferential testing, data distribution normality was verified using the Shapiro–Wilk test (shapiro.test function, stats package), and homogeneity of variance was evaluated using Levene’s test in GraphPad Prism software (version 8.0; GraphPad Software, San Diego, CA, USA).

For longitudinal datasets evaluating the effects of treatment across developmental time points (e.g., weekly sickness behaviors, body weights, and temporal copulatory metrics), a two-way unrepeated-measures Analysis of Variance (Two-Way ANOVA) was conducted to evaluate the main effects of Treatment, Time, and their interaction (Treatment X Time). When significant interaction effects were detected, Tukey’s post-hoc test was applied for multiple pairwise comparisons.

For single-endpoint datasets including terminal reproductive data (maturity age and weight), and DNA damage parameters (hepatic and cerebral Comet assay indicators) unpaired independent Student’s *t*-tests were employed to evaluate statistical differences between control and imidacloprid-treated groups (*n* = 5 per sex per group). In all statistical evaluations, differences were considered statistically significant at a threshold of *p* < 0.05. All experimental data are expressed as the mean ± standard error of the mean (SEM).

## 3 Results

### 3.1 Both sex obtained data

#### 3.1.1 Genotoxicity markers

Assessment of genotoxicity via the alkaline comet assay demonstrated that imidacloprid exposure induced significant DNA damage in both the hepatic and cerebral tissues of quails compared to the control group (Figure 1). In liver tissue, imidacloprid administration resulted in a marked elevation in the percentage of DNA damage (*p* < 0.001), tail length (*p* < 0.05), and tail moment (*p* < 0.001), while percentage of DNA in the tail and Olive tail moment showed no statistically significant differences. Similarly, brain tissue from imidacloprid-treated quails exhibited a pronounced increase in the percentage of DNA damage (*p* < 0.001) and tail moment (*p* < 0.05) relative to controls, whereas tail length, DNA in tail, and Olive tail moment remained without significant alteration (Supplementary Figures 4, 5).

**Figure 1:**
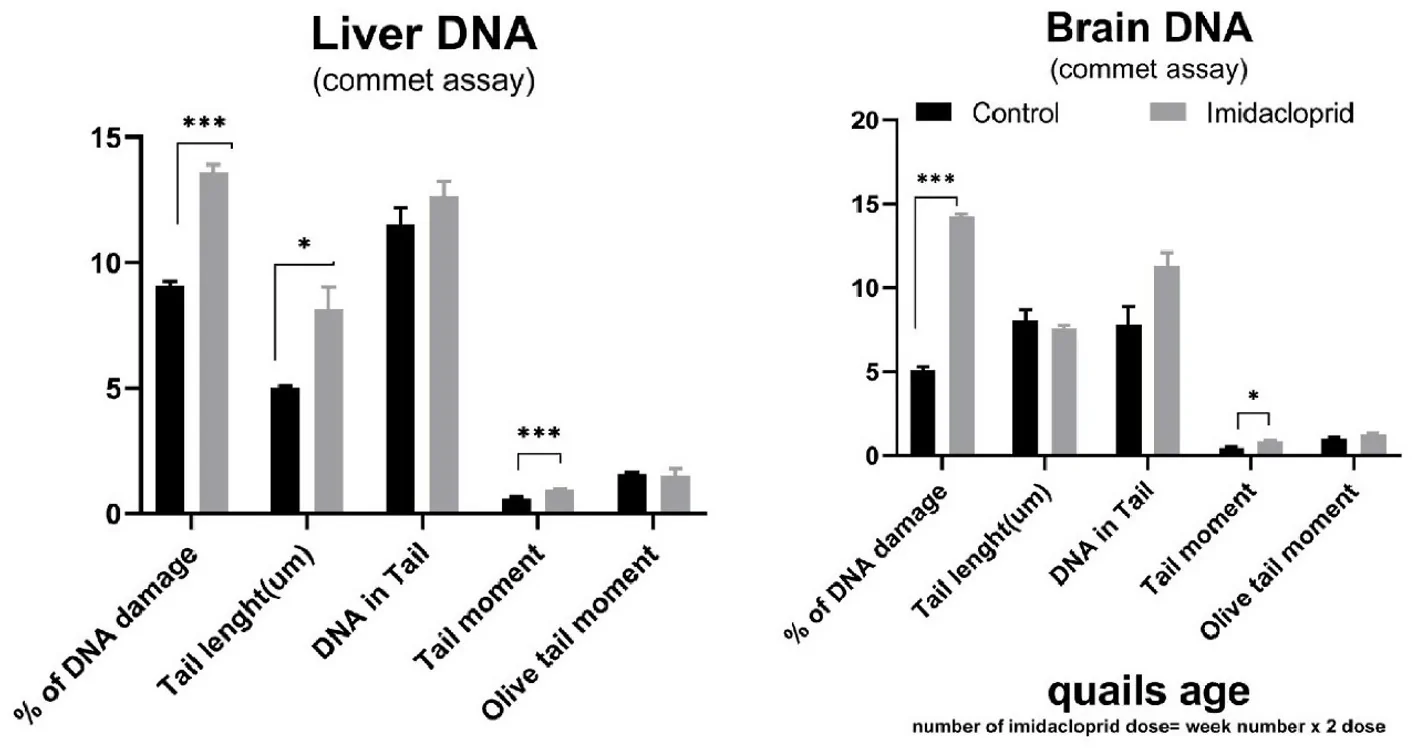
Effects of chronic imidacloprid exposure on DNA damage in liver and brain tissues of Japanese quail (Coturnix coturnix japonica) across weeks 7 to 12 of age. Data are presented as mean ± SE. Asterisks denote statistically significant differences between control and imidacloprid-treated groups at individual weeks: *P < 0.05, *P* < 0.01, ***P < 0.001, ****P < 0.0001.

### 3.2 Endocrine profiles and gonadal hormone levels

Figure 2 show that, chronic exposure to imidacloprid significantly disrupted circulating sex steroid and gonadotropin concentrations in Japanese quail across weeks 7 to 12 of age. Circulating testosterone was profoundly suppressed by imidacloprid, showing significant main effects of treatment (F_1, 48_ = 88.84, *p* < 0.0001) and time [*F*(5, 48) = 316.9, *p* < 0.0001], alongside a significant treatment x time interaction [*F*(5, 48) = 5.390, *p* = 0.0005], with post-hoc multiple comparisons revealing marked reductions in treated birds at Week 10 (*p* < 0.001) and Week 11 (*p* < 0.0001). Total estrogens followed a similar inhibitory trend [treatment: *F*(1, 48) = 198.5, *p* < 0.0001; time: *F*(5, 48) = 1898, *p* < 0.0001; interaction: *F*(5, 48) = 5.000, *p* = 0.0009], with significant depletion occurring at Week 7 (*p* < 0.01), Week 10 (*p* < 0.001), Week 11 (*p* < 0.0001), and Week 12 (*p* < 0.0001). Progesterone levels were consistently reduced in exposed quails [treatment: *F* (1, 48) = 47.10, *p* < 0.0001; time: *F*(5, 48) = 13.84, *p* < 0.0001; interaction: *F*(5, 48) = 1.188, *p* = 0.3289], with a significant reduction observed at Week 7 (*p* < 0.01). Levels of 17beta-estradiol displayed significant main effects of treatment [*F*(1, 48) = 74.22, *p* < 0.0001] and time [*F*(5, 48) = 208.6, *p* < 0.0001] along with an interaction effect [*F*(5, 48) = 3.302, *p* = 0.0121)], characterized by an initial increase at Week 7 (*p* < 0.01) followed by sharp suppression at Week 9 (*p* < 0.0001).

**Figure 2:**
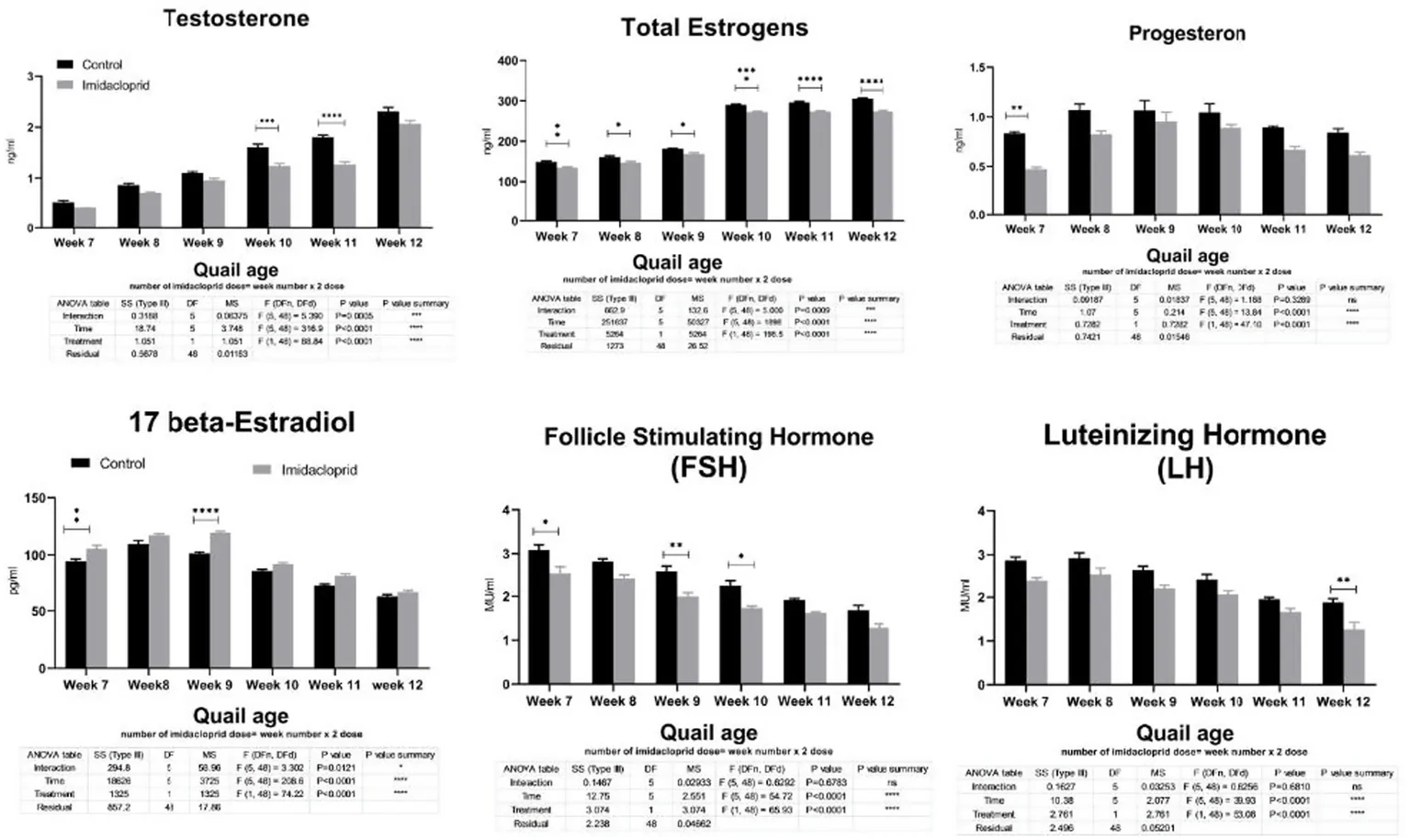
Effects of chronic imidacloprid exposure on circulating sex steroid and gonadotropin concentrations in Japanese quail (Coturnix coturnix japonica) across weeks 7 to 12 of age. Data are represented as mean ± SEM. Statistical differences between the control and imidacloprid-treated groups at each time point are indicated by asterisks: (*P < 0.05, *P* < 0.01, ***P < 0.001, ****P < 0.0001; ns = non-significant).

Furthermore, anterior pituitary gonadotropins were significantly diminished; follicle-stimulating hormone (FSH) was depressed across treatment [*F*(1, 48) = 65.93, *p* < 0.0001] and time [*F*(5, 48) = 54.72, *p* < 0.0001; interaction: *F*(5, 48) = 0.6292, *p* = 0.6783], exhibiting significant weekly declines at Week 7 (*p* < 0.05), Week 9 (*p* < 0.01), and Week 10 (*p* < 0.05), while luteinizing hormone (LH) was similarly inhibited [treatment: *F*(1, 48) = 53.08, *p* < 0.0001; time: *F*(5, 48) = 39.93, *p* < 0.0001; interaction: *F*(5, 48) = 0.6256, *p* = 0.6810], culminating in a significant reduction at Week 12 (*p* < 0.01), collectively demonstrating systemic disruption of the hypothalamic–pituitary-gonadal (HPG) axis.

### 3.3 Sexual maturity parameters

Exposure to imidacloprid significantly delayed the onset of sexual maturation and increased body weight at sexual maturity in both male and female quails (Table 1). Compared to the control group, imidacloprid-treated males reached reproductive maturity significantly later (44.22 days vs. 41.9 days; *p* = 0.001) and at a significantly higher body weight (149.55 vs. 145.7; *p* = 0.01). Similarly, female quails exposed to imidacloprid exhibited a significant delay in the age of reproductive maturity (47.44 days vs. 45.3 days; *p* = 0.003) alongside a marked increase in body weight at maturity (191.89 vs. 182.9 g; *p* = 0.001) relative to untreated controls.

**Table 1:** Effect of imidacloprid exposure on the age (days) and body weight (g) at reproductive maturity in male and female quails.

	Control	Imidacloprid	P value
Reproductive maturity age (days)			
Male quails	41.9 ± 0.42	44.22 ± 0.41	0.001
Female quails	45.3 ± 0.50	47.44 ± 0.34	0.003
Reproductive maturity weight (gram)			
Male quails	145.7 ± 1.10	149.55 ± 0.69	0.01
Female quails	182.9 ± 1.63	191.89 ± 1.73	0.001

Data are presented as mean ± standard error (SE). Differences between the control and imidacloprid groups were considered statistically significant at *P* < 0.05 and p ≤ 0.01.

### 3.4 Brain somatic and gonadosomatic indices (BSI and GSI)

Two-way ANOVA revealed that subchronic imidacloprid exposure caused severe, sexually dimorphic systemic somatic and gonadal organ mass reductions in Japanese quail (*Coturnix coturnix japonica*) across developmental ages (Weeks 7–12) (Figure 3).

**Figure 3:**
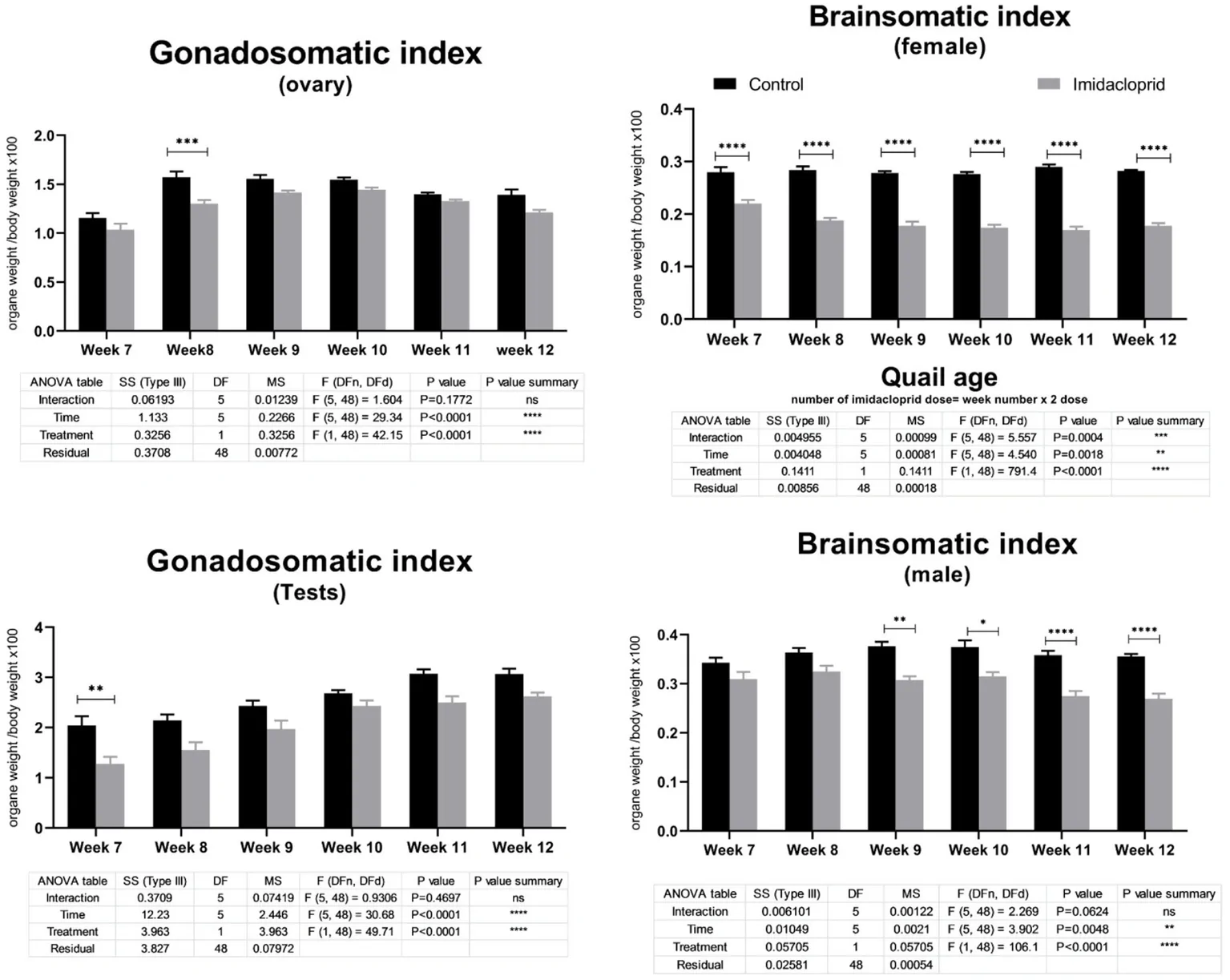
Somatometric organ alterations induced by subchronic imidacloprid exposure in male and female Japanese quail (Coturnix coturnix japonica) across developmental stages (Weeks 7–12). Data are represented as mean ± SEM. Statistical differences between the control and imidacloprid-treated groups at each time point are indicated by asterisks: (*P < 0.05, *P* < 0.01, ***P < 0.001, ****P < 0.0001; ns = non-significant).

The female brainsomatic index (BSI) was profoundly depressed following imidacloprid administration [*F*(1, 48) = 791.40, *p* < 0.0001], displaying a significant main effect of age [*F*(5, 48) = 4.540, *p* = 0.0018] and a highly significant treatment X Times age interaction [*F*(5, 48) = 5.557, *p* = 0.0004], with persistent, highly significant reductions across all observation intervals from Week 7 through Week 12 (*p* < 0.0001).

In males, BSI was likewise significantly reduced by treatment [*F*(1, 48) = 106.10, *p* < 0.0001] and influenced by age [*F*(5, 48) = 3.902, *p* = 0.0048; interaction: *F*(5, 48) = 2.269, *p* = 0.0624], with post-hoc comparisons showing prominent organ-to-body mass deficits at Week 9 (*p* < 0.01), Week 10 (*p* < 0.05), Week 11 (*p* < 0.0001), and Week 12 (*p* < 0.0001).

Evaluation of gonadal indices demonstrated marked gonadal hypotrophy in both sexes: the female ovarian gonadosomatic index (GSI) was significantly suppressed [*F*(1, 48) = 42.15, *p* < 0.0001; time: *F* (5, 48) = 29.34, *p* < 0.0001; interaction: *F* (5, 48) = 1.604, *p* = 0.1772], highlighted by a prominent reduction at Week 8 (*p* < 0.001), while the male testicular GSI was severely inhibited by imidacloprid [*F*(1, 48) = 49.71, *p* < 0.0001; time: *F* (5, 48) = 30.68, *p* < 0.0001; interaction: *F* (5, 48) = 0.9306, *p* = 0.4697], with significant testicular mass deficits starting early at Week 7 (*p* < 0.01).

### 3.5 General and sickness behavioral alterations

Chronic oral administration of imidacloprid significantly disrupted normal maintenance and comfort behaviors in Japanese quail from weeks 7 to 12 of age (Figure 4). For feeding behavior, two-way ANOVA revealed significant main effects of treatment [*F*(1, 24) = 35.76, *p* < 0.0001] and time [*F*(5, 24) = 7.816, *p* = 0.0002] without a significant interaction [*F*(5, 24) = 0.5009, *p* = 0.7725], with treated birds showing generally higher feeding frequencies early on before decreasing alongside controls by week 12.

**Figure 4:**
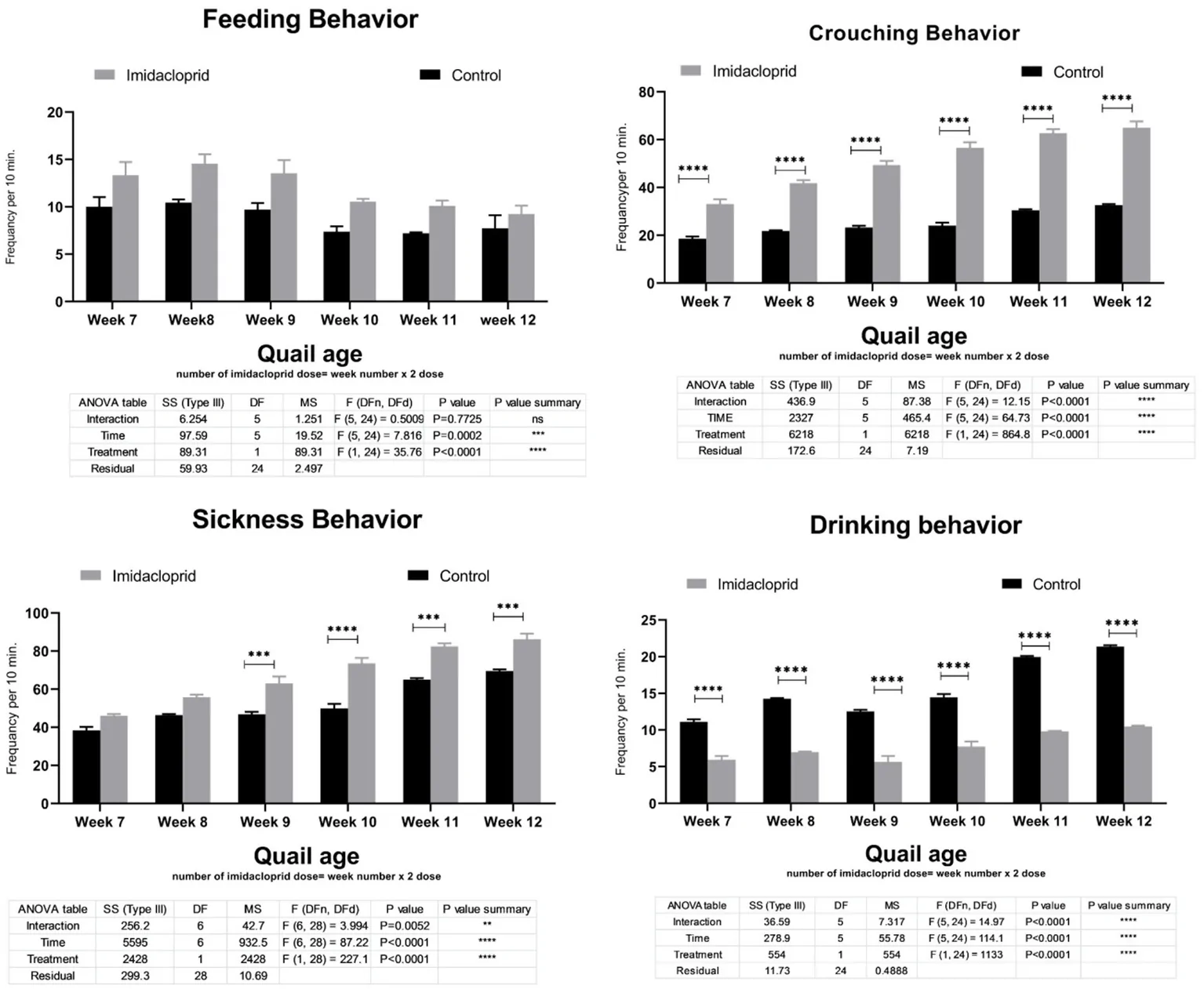
Effects of chronic imidacloprid exposure on the behavioral patterns of Japanese quail (*Coturnix coturnix japonica*) from weeks 7 to 12 of age. Data are expressed as mean ± SEM. Statistical differences between the control and imidacloprid-treated groups at each time point are indicated by asterisks: * *P* < 0.01, ***P < 0.001, ****P < 0.0001.

In contrast, crouching behavior exhibited a pronounced, progressive increase in imidacloprid-treated birds, supported by significant effects of treatment [*F*(1, 24) = 864.8, *p* < 0.0001], time [*F*(5, 24) = 64.73, *p* < 0.0001], and their interaction [*F*(5, 24) = 12.15, *p* < 0.0001], with post-hoc tests indicating significantly elevated crouching at all-time points (*p* < 0.0001). Similarly, sickness behavior increased substantially over time, showing significant main effects of treatment [*F*(1, 28) = 227.1, *p* < 0.0001] and time [*F*(6, 28) = 87.22, *p* < 0.0001] along with a significant interaction [*F*(6, 28) = 3.994, *p* = 0.0052], where treated birds manifested markedly higher sickness frequencies from Week 9 through Week 12 (*p* < 0.001). Conversely, drinking behavior was severely suppressed across the entire exposure period [treatment: *F* (1, 24) = 1,133, *p* < 0.0001; time: *F*(5, 24) = 114.1, *p* < 0.0001; interaction: *F*(5, 24) = 14.97, *p* < 0.0001], with treated quails displaying a continuous, significant reduction in drinking bouts compared to controls at every evaluated week (*p* < 0.0001).

## 4 Male-specific reproductive and behavioral parameters

### 4.1 Male reproductive parameters

Regarding male reproductive organs and fertility markers, imidacloprid administration, bird age (time), and their interaction significantly influenced cloacal gland area (CAREA), relative testis weight, and foam production (Table 2).

**Table 2:** Effect of imidacloprid treatment, time (weeks 7–12), and their interaction on cloacal gland area (CAREA), relative testis weight, and foam weight in male quails.

	(CAREA) (mm^2^)	Relative testis weight	Foam weight (mg)
Treatment			
Control	332.9	0.03	58.5
Imidacloprid	304.66	0.02	50.06
SE	0.399	0.001	0.229
P value	0.0000	0.000	0.000
Time			
Week 7	201.5	0.016	19.5
Week 8	273.5	0.027	41.6
Week 9	320.17	0.027	48
Week 10	340	0.029	57
Week 11	358.5	0.031	74.5
Week 12	419.17	0.034	85
SE	0.661	0.0001	0.379
P value	0.0000	0.000	0.000
Treatment x time interaction			
Week 7 control	209	0.022	23
Week 8 control	285.3	0.034	45
Week 9 control	334	0.034	51
Week 10 control	356	0.034	61
Week 11 control	382	0.036	80
Week 12 control	431.33	0.037	91
Week 7 treatment	194	0.01	16
Week treatment	261.66	0.02	38.33
Week 9 treatment	306.33	0.02	45
Week 10 treatment	324	0.023	53
Week 11 treatment	335	0.027	69
Week 12 treatment	407	0.030	79
SE	0.997	0.002	0.561
*P* value	0.0000	0.000	0.000

Data are presented as mean ± standard error (SE). Statistical significance was assessed across main effects (treatment and time) and treatment x time interaction (*P* < 0.001).

Overall, imidacloprid exposure caused a highly significant reduction in mean cloacal gland area (304.66 vs. 332.90 mm), relative testis weight (0.02 vs. 0.03), and foam weight (50.06 vs. 58.50 mg) compared to the control group (*p* < 0.001 for all). While all measured reproductive metrics increased progressively with age from week 7 to week 12 (*p* < 0.001), imidacloprid-treated males consistently exhibited significantly lower values for cloacal gland area, relative testis weight, and foam weight at every weekly time point compared to time-matched controls (*p* < 0.001).

### 4.2 Sexual and active courtship behaviors (within the rearing cage)

Figure 5 illustrated that, chronic administration of imidacloprid significantly suppressed reproductive and active Appetitive (courtship) behaviors in male Japanese quail across weeks 7 to 12 of age. For absolute chase frequency, two-way ANOVA revealed significant main effects of treatment [*F*(1, 48) = 139.0, *p* < 0.0001] and time [*F*(5, 48) = 2.747, *p* = 0.0292] without a significant interaction [*F*(5, 48) = 0.9929, *p* = 0.4321], with post-hoc analysis demonstrating a persistent reduction in chasing events in treated quail across all evaluated weeks (*p* < 0.05 to *p* < 0.0001). Similarly, absolute waltzing frequency was significantly inhibited in imidacloprid-treated males, exhibiting main effects of treatment [*F*(1, 48) = 99.23, *p* < 0.0001] and time [*F*(5, 48) = 34.80, *p* < 0.0001] along with a significant interaction [*F*(5, 48) = 3.450, *p* = 0.0096], with marked reductions emerging from Week 9 through Week 12 (*p* < 0.05 to *p* < 0.0001). Wing flapping frequency also declined sharply following exposure, confirmed by significant effects of treatment [*F*(1, 48) = 98.53, *p* < 0.0001] and treatment x time interaction [*F*(5, 48) = 3.743, *p* = 0.0061], with significant post-hoc suppression evident from Week 9 through Week 12 (*p* < 0.01 to *p* < 0.0001). In contrast, analysis of behavioral proportions relative to total activity revealed no significant main effect of treatment or interaction for chase proportion [*F*(1, 48) = 1.168, *p* = 0.2851], waltzing proportion [*F*(1, 48) = 0.03304, *p* = 0.8565], or wing flapping proportion [*F*(1, 48) = 2.452, *p* = 0.1240], demonstrating that the observed reduction in sexual and courtship displays was primarily driven by generalized behavioral depression and overall hypoactivity rather than selective shifts in relative behavioral allocation.

**Figure 5:**
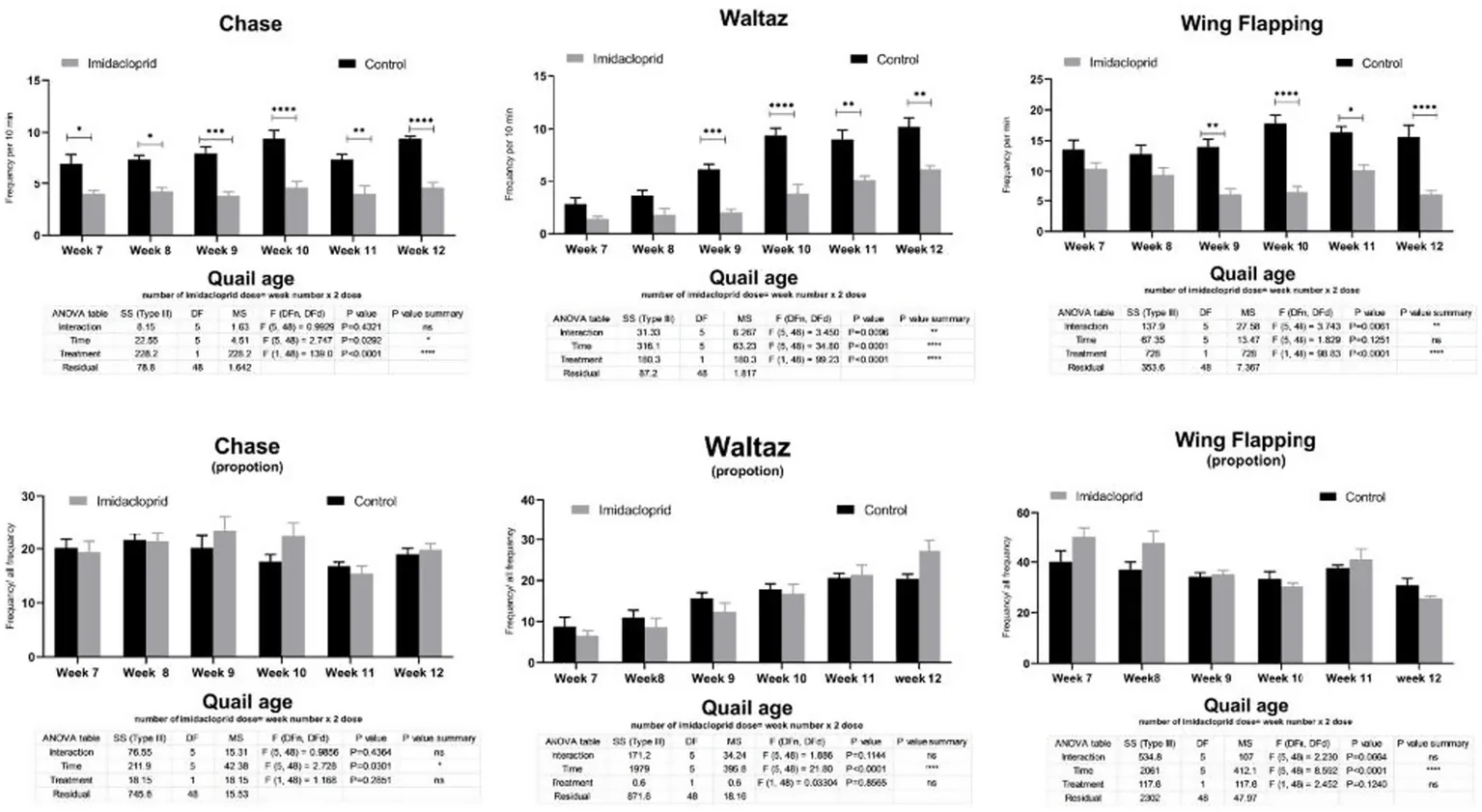
Effects of chronic imidacloprid exposure on Appetitive (courtship) and active behavioral patterns in male Japanese quail (*Coturnix coturnix japonica*) from weeks 7 to 12 of age. Data are expressed as mean ± SEM. Statistical differences between the control and imidacloprid-treated groups at each time point are indicated by asterisks: * *P* < 0.01, ***P < 0.001, ****P < 0.0001.

### 4.3 Copulatory and mating behaviors (rearing cages and testing arena)

Figures 6, 7 illustrated that, chronic oral exposure to imidacloprid consistently suppressed consummatory copulatory behaviors in male Japanese quail across weeks 7 to 12 of age, both in home rearing cages and within the standard behavioral testing arena. Inside home cages, significant main effects of treatment (*p* < 0.0001) and time (*p* < 0.05) without interactions were observed across all primary mating activities, resulting in marked reductions in the absolute frequencies of neck grabbing, mounting, and successful cloacal contact (*p* < 0.05 to *p* < 0.0001). Proportional behavioral analysis in the home cages further revealed a significant suppression in cloacal contact [*F*(1, 48) = 38.59, *p* < 0.0001] and mounting proportions [*F*(1, 48) = 9.246, *p* = 0.0038]. These impairments were fully corroborated in the standard testing arena, where treated males exhibited significant reductions in absolute mount frequency [*F*(1, 48) = 15.75, *p* = 0.0002], cloacal contacts [*F*(1, 48) = 9.377, *p* = 0.0036], neck grabs [*F*(1, 48) = 7.811, *p* = 0.0074], and mount attempts [*F*(1, 48) = 13.44, *p* = 0.0006], alongside a selective decrease in relative cloacal contact proportion [*F*(1, 48) = 4.762, *p* = 0.0340]. Together, these concordant findings indicate that while imidacloprid causes a generalized suppression across all absolute mating activities, it selectively impairs the motor proficiency and consummatory efficiency necessary to progress from appetitive mounting to successful cloacal contact.

**Figure 6:**
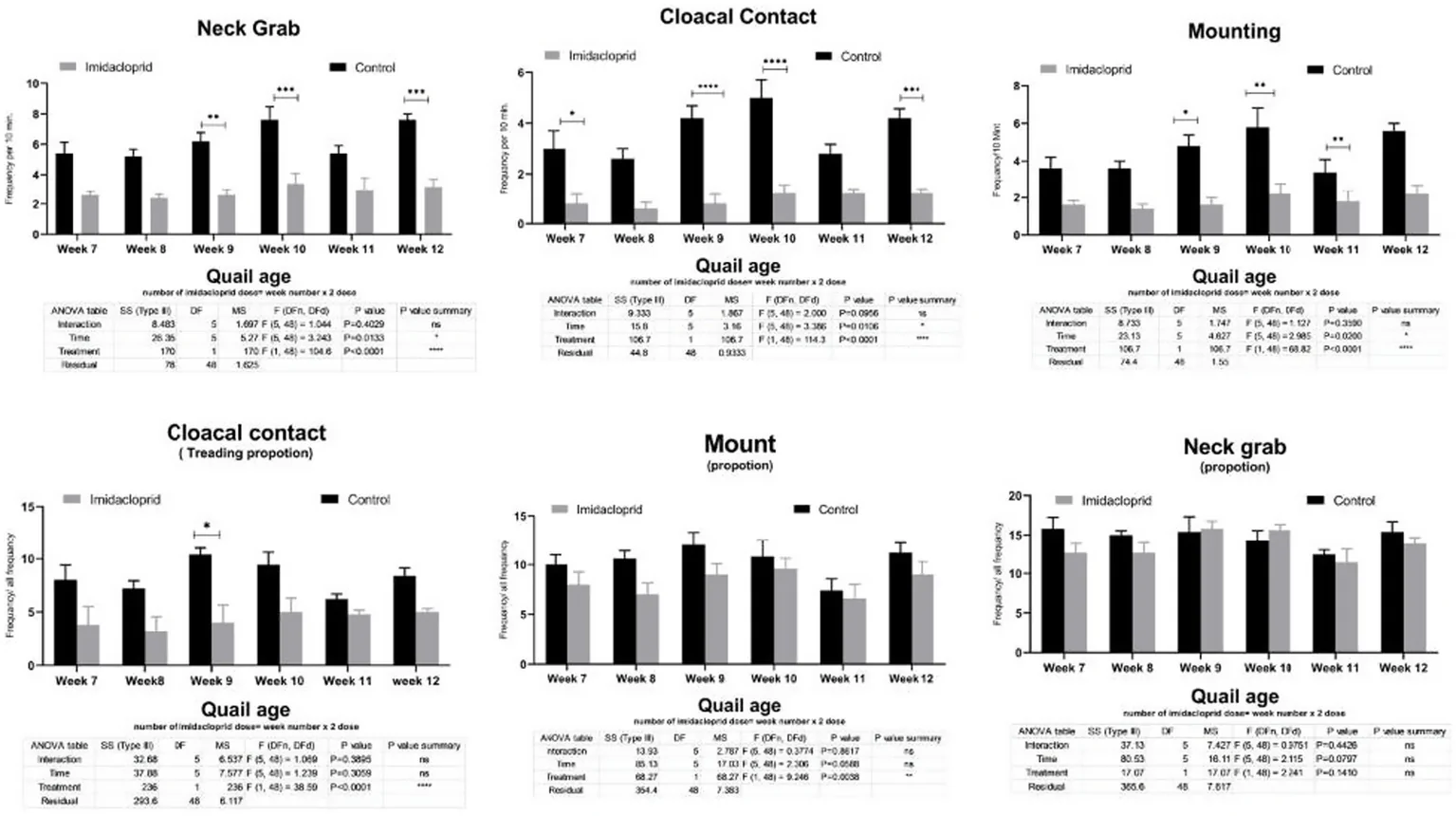
Effects of subchronic imidacloprid exposure on consummatory copulatory behaviors in male Japanese quail (Coturnix coturnix japonica) across weeks 7 to 12 of age inside rearing cage. Data are expressed as mean ± SEM. Statistical differences between the control and imidacloprid-treated groups at each time point are indicated by asterisks: *P < 0.05, ** *P* < 0.01, ***P < 0.001, ****P < 0.0001.

**Figure 7:**
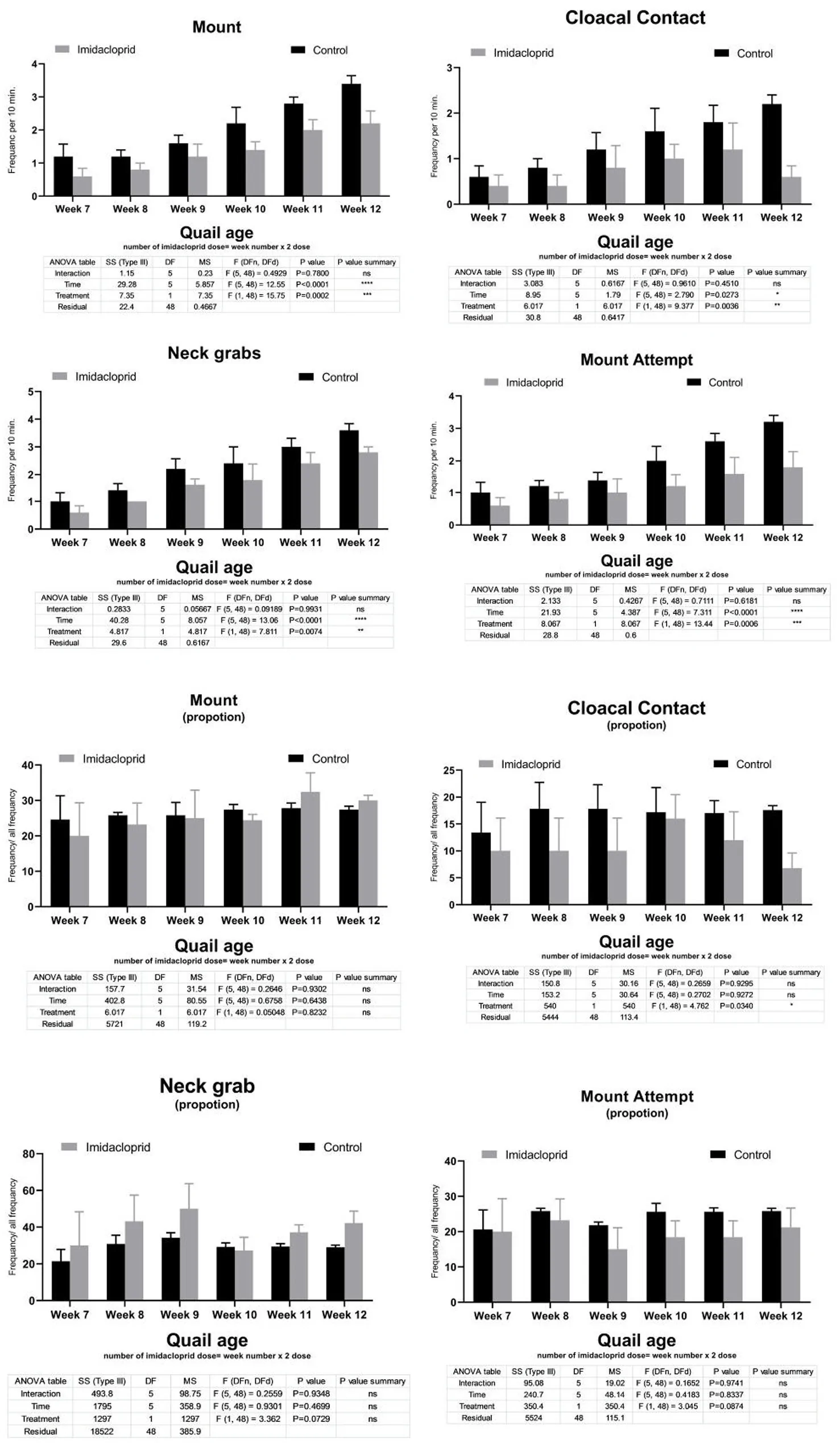
Effects of subchronic imidacloprid exposure on consummatory copulatory behaviors in male Japanese quail (Coturnix coturnix japonica) across weeks 7 to 12 of age inside test cage. Data are expressed as mean ± SEM. Ns mean no significant difference.

### 4.4 Male behavioral evaluations in an external testing arena

#### 4.4.1 Sexual motivation, latencies, and libido scoring

During the initial 2-min screening window in an external arena, (Figure 8) chronic administration of imidacloprid significantly disrupted sexual motivation and temporal dynamics during mating encounters in male Japanese quail from weeks 7 to 12 of age. For latency to first sexual interaction, two-way ANOVA revealed significant main effects of treatment [*F*(1, 48) = 50.50, *p* < 0.0001] and time [*F*(5, 48) = 100.3, *p* < 0.0001] without a significant interaction [*F*(5, 48) = 1.863, *p* = 0.1185], with treated males showing significantly prolonged initial latencies during earlier maturity at Week 7 (*p* < 0.01) and Week 8 (*p* < 0.01).

**Figure 8:**
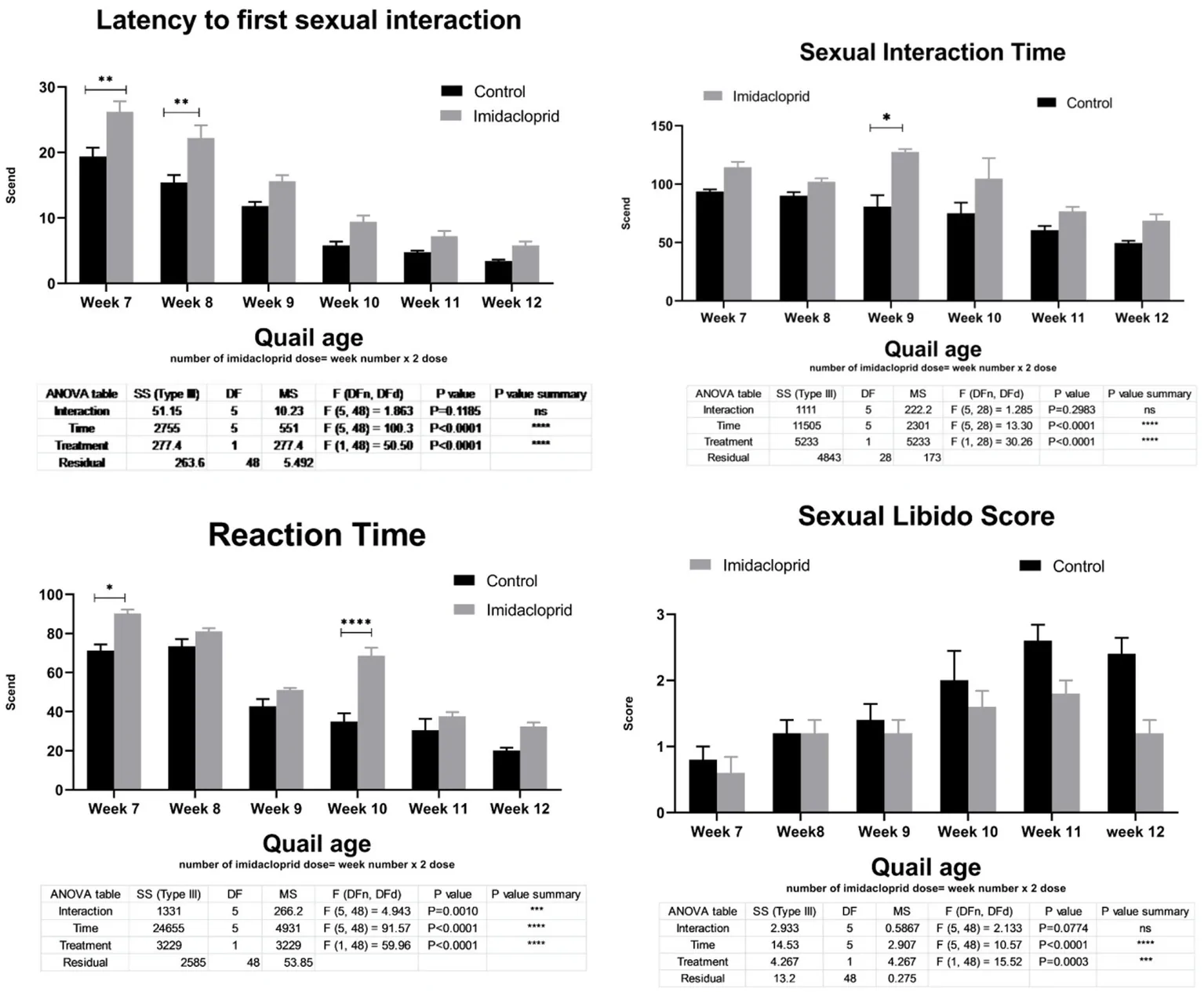
Effects of chronic imidacloprid exposure on sexual motivation, latencies, and libido scoring in male Japanese quail (Coturnix coturnix japonica) in the initial two mint in testing box across weeks 7 to 12 of age. Data are expressed as mean ± SEM. Statistical differences between the control and imidacloprid-treated groups at each time point are indicated by asterisks: *P < 0.05, ***P < 0.001, ****P < 0.0001.

Similarly, total sexual interaction time demonstrated significant main effects of treatment [*F*(1, 28) = 30.26, *p* < 0.0001] and time [*F*(5, 28) = 13.30, *p* < 0.0001; interaction: *F*(5, 28) = 1.285, *p* = 0.2983], with treated birds exhibiting extended duration spent attempting interactions, marked by a significant increase at Week 9 (*p* < 0.05).

Reaction time was also markedly impaired following imidacloprid exposure, supported by significant main effects of treatment [*F*(1, 48) = 59.96, *p* < 0.0001] and time [*F*(5, 48) = 91.57, *p* < 0.0001], alongside a significant treatment x Time interaction [*F*(5, 48) = 4.943, *p* = 0.0010], with post-hoc tests indicating substantially delayed response times in exposed birds at Week 7 (*p* < 0.05) and Week 10 (*p* < 0.0001). Correspondingly, the sexual libido score was significantly depressed in the imidacloprid group, confirmed by significant main effects of treatment [*F*(1, 48) = 15.52, *p* = 0.0003] and time [*F*(5, 48) = 10.57, *p* < 0.0001; interaction: *F*(5, 48) = 2.133, *p* = 0.0774], reflecting an overall attenuation in sexual drive and courtship responsiveness across development compared to control counterparts.

#### 4.4.2 Mating success and copulatory efficiency

Figure 9 chronic oral exposure to imidacloprid significantly compromised mating success in male Japanese quail across weeks 7 to 12 of age. For successful mating frequency, two-way ANOVA demonstrated highly significant main effects of treatment [*F*(1, 48) = 114.3, *p* < 0.0001] and time [*F*(5, 48) = 3.386, *p* = 0.0106], while the interaction between treatment and time was not statistically significant [*F*(5, 48) = 2.000, *p* = 0.0956].

**Figure 9:**
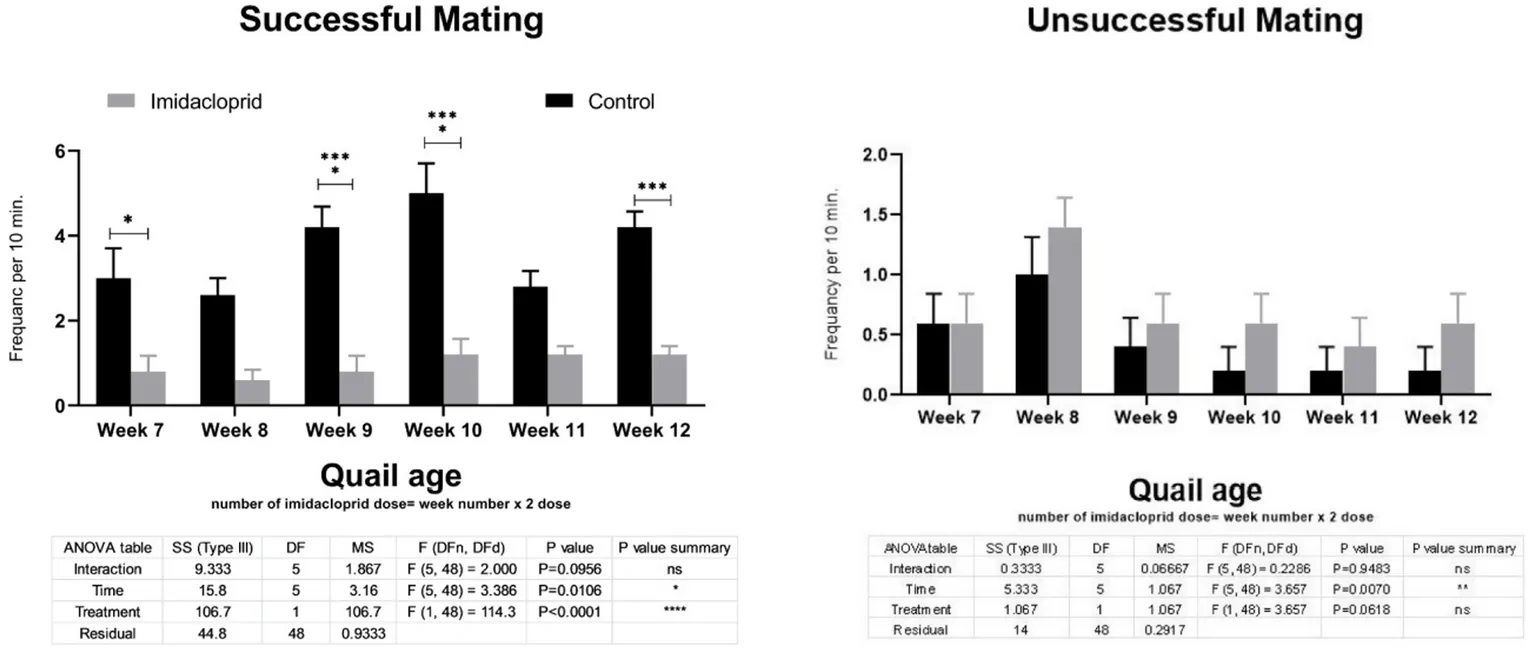
Effects of chronic imidacloprid exposure on mating success and efficiency in male Japanese quail (*Coturnix coturnix japonica*) across weeks 7 to 12 of age. Data are expressed as mean ± SEM. Statistical differences between the control and imidacloprid-treated groups at each time point are indicated by asterisks: *P < 0.05, ** *P* < 0.01, ***P < 0.001, ****P < 0.0001.

Imidacloprid-treated males exhibited a pronounced and sustained deficit in completing successful matings compared to controls, with post-hoc multiple comparisons confirming significant reductions at Week 7 (*p* < 0.05), Week 9 (*p* < 0.001), Week 10 (*p* < 0.0001), and Week 12 (*p* < 0.001). In contrast, unsuccessful mating frequency showed a significant main effect of time [*F*(5, 48) = 3.657, *p* = 0.0070], but no significant main effect of treatment [*F*(1, 48) = 3.657, *p* = 0.0618] or treatment x time interaction [*F*(5, 48) = 0.2286, *p* = 0.9483], indicating that the neurotoxic burden of imidacloprid selectively depresses copulatory competence and successful copulatory completion rather than merely elevating aborted or incomplete mating attempts.

### 4.5 Mating performance, sexual experience indices, and copulatory efficiency

Figure 10 show that, chronic oral exposure to imidacloprid significantly compromised sexual proficiency, behavioral indices, and copulatory efficiency in male Japanese quail from weeks 7 to 12 of age. The sexual experience index exhibited significant main effects of treatment [*F*(1, 28) = 30.17, *p* < 0.0001] and time [*F*(5, 28) = 25.04, *p* < 0.0001], alongside a significant treatment x time interaction [*F*(5, 28) = 3.229, *p* = 0.0200], with exposed males displaying abnormally prolonged index values at Week 10 (*p* < 0.05) and Week 12 (*p* < 0.01) reflective of protracted, inefficient sexual interactions [treatment: *F*(1, 28) = 30.26, *p* < 0.0001; time: *F*(5, 28) = 13.30, *p* < 0.0001; Week 9: *p* < 0.05]. Consistently, absolute cloacal contact movements were significantly suppressed by imidacloprid [treatment: *F* (1, 48) = 9.377, *p* = 0.0036; time: *F*(5, 48) = 2.790, *p* = 0.0273], with a marked reduction reaching significance by Week 12 (*p* < 0.05). Semi-quantitative behavioral scores similarly revealed severe impairment, as treated males demonstrated highly significant reductions in mount score [treatment: *F*(1, 48) = 41.78, *p* < 0.0001; time: *F*(5, 48) = 2.443, *p* = 0.0473], mount attempt score [treatment: *F*(1, 48) = 35.51, *p* < 0.0001; Week 9: *p* < 0.05], and positive cloacal contact score [treatment: *F*(1, 24) = 96.00, *p* < 0.0001; time: *F*(5, 24) = 24.00, *p* < 0.0001; interaction: *F*(5, 24) = 6.000, *p* = 0.0010], the latter showing marked suppression across weeks 10, 11, and 12 (*p* < 0.001). Finally, copulatory efficiency (calculated as cloacal contacts per neck grab) inside the home rearing cages was substantially depleted in treated birds [treatment: *F*(1, 47) = 28.80, *p* < 0.0001; time: *F*(5, 47) = 8.173, *p* < 0.0001; Week 9: *p* < 0.05], an inhibitory trend that was similarly paralleled within the testing box [treatment: *F*(1, 45) = 3.879, *p* = 0.0551], collectively confirming that imidacloprid disrupts both appetitive courtship motivation and consummatory copulatory competence.

**Figure 10:**
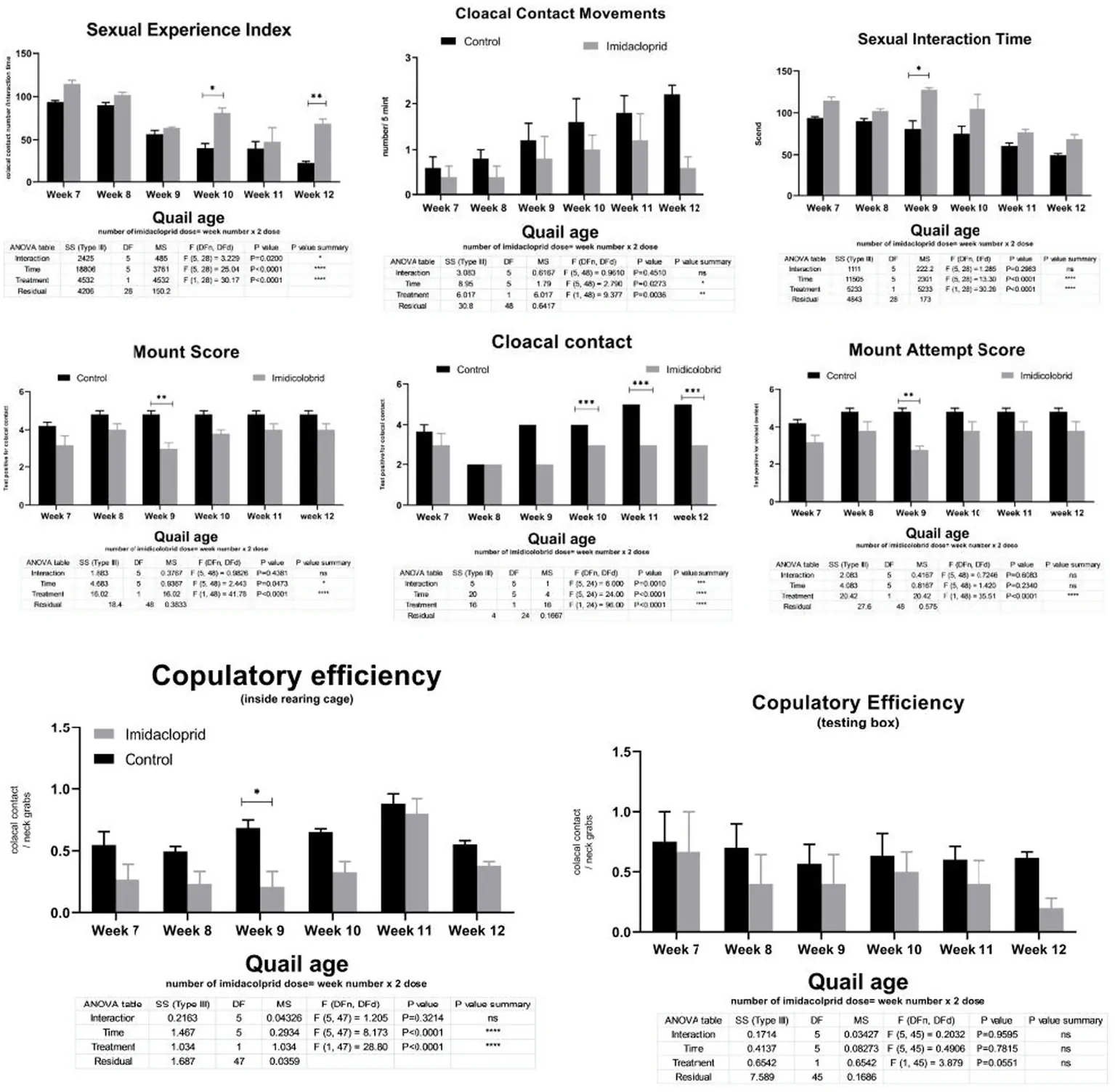
Effects of chronic imidacloprid exposure on sexual performance indices, sexual experience index and copulatory efficiency in male Japanese quail (Coturnix coturnix japonica) across weeks 7 to 12 of age. Data are expressed as mean ± SEM. Statistical differences between the control and imidacloprid-treated groups at each time point are indicated by asterisks: *P < 0.05, ***P < 0.001, ****P < 0.0001.

## 5 Female-specific reproductive and behavioral parameters

### 5.1 Female reproductive performance indices

Assessment of female reproductive performance demonstrated that imidacloprid exposure, age (time), and their interaction exerted a highly significant adverse effect on total weekly egg production; egg weight, hen-day egg production (HDEP), and egg mass (Table 3).

**Table 3:** Effect of imidacloprid administration, time (weeks 7–12), and their interaction on total egg number per week, egg weight (g), hen-day egg production (HDEP, %), and egg mass in female quails.

	Total egg number per week	Egg weight (gram)	HDEP	Egg mass
Treatment				
Control	36.8	14.7	72.7	0.4
Imidacloprid	31.7	11.3	62.4	0.13
SE	0.2	0.16	0.184	0.05
P value	0.000	0.000	0.000	0.001
Time				
Week 7	37.5	11	52.67	0.36
Week 8	37	12.8	57.83	0.49
Week 9	35.7	13.17	63.67	0.24
Week 10	34.2	13.5	70.83	0.10
Week 11	32.8	13.7	77.5	0.26
Week 12	28.7	14	83	0.12
SE	0.347	0.272	0.319	0.09
*P* value	0.000	0.000	0.000	0.03
Treatment x time interaction				
Week 7 control	41	11.7	57	0.67
Week 8 control	40	15	63.3	0.92
Week 9 control	38	15	68	0.40
Week 10 control	36	15.3	74.7	0.12
Week 11 control	35.3	15.7	83.3	0.13
Week 12 control	31	15.7	90	0.15
Week 7 treatment	34	10.3	48.3	0.05
Week treatment	34	10.7	52.3	0.06
Week 9 treatment	33.3	11.3	59.3	0.07
Week 10 treatment	32.3	11.7	67	0.08
Week 11 treatment	30.3	11.7	71.7	0.4
Week 12 treatment	26.3	12.3	76	0.09
SE	0.491	0.385	0.451	0.122
*P* value	0.000	0.000	0.000	0.001

Data represent mean ± standard error (SE). Statistical significance was assessed across main factors (treatment, time) and the treatment x time interaction (*P* < 0.05, *P* < 0.001).

Overall, imidacloprid administration led to a substantial reduction in total egg number per week (31.7 vs. 36.8), egg weight (11.3 vs. 14.7 g), HDEP (62.4 vs. 72.7), and egg mass (0.13 vs. 0.40) compared to the control group (*p* < 0.001 for all). Across the laying period from week 7 to week 12, egg weight and HDEP significantly increased with age, whereas total weekly egg numbers gradually declined (*p* < 0.001). Furthermore, the treatment x time interaction was statistically significant for all measured traits (*p* ≤ 0.001), with the imidacloprid-treated hens consistently producing fewer, lighter eggs and exhibiting markedly depressed HDEP and egg mass at every weekly interval relative to their time-matched controls.

### 5.2 Female sexual receptivity, mounting responses, and egg laying behavior

Figure 11 show that, chronic administration of imidacloprid significantly suppressed sexual receptivity and reproductive output in female Japanese quail across weeks 7 to 12 of age.

**Figure 11:**
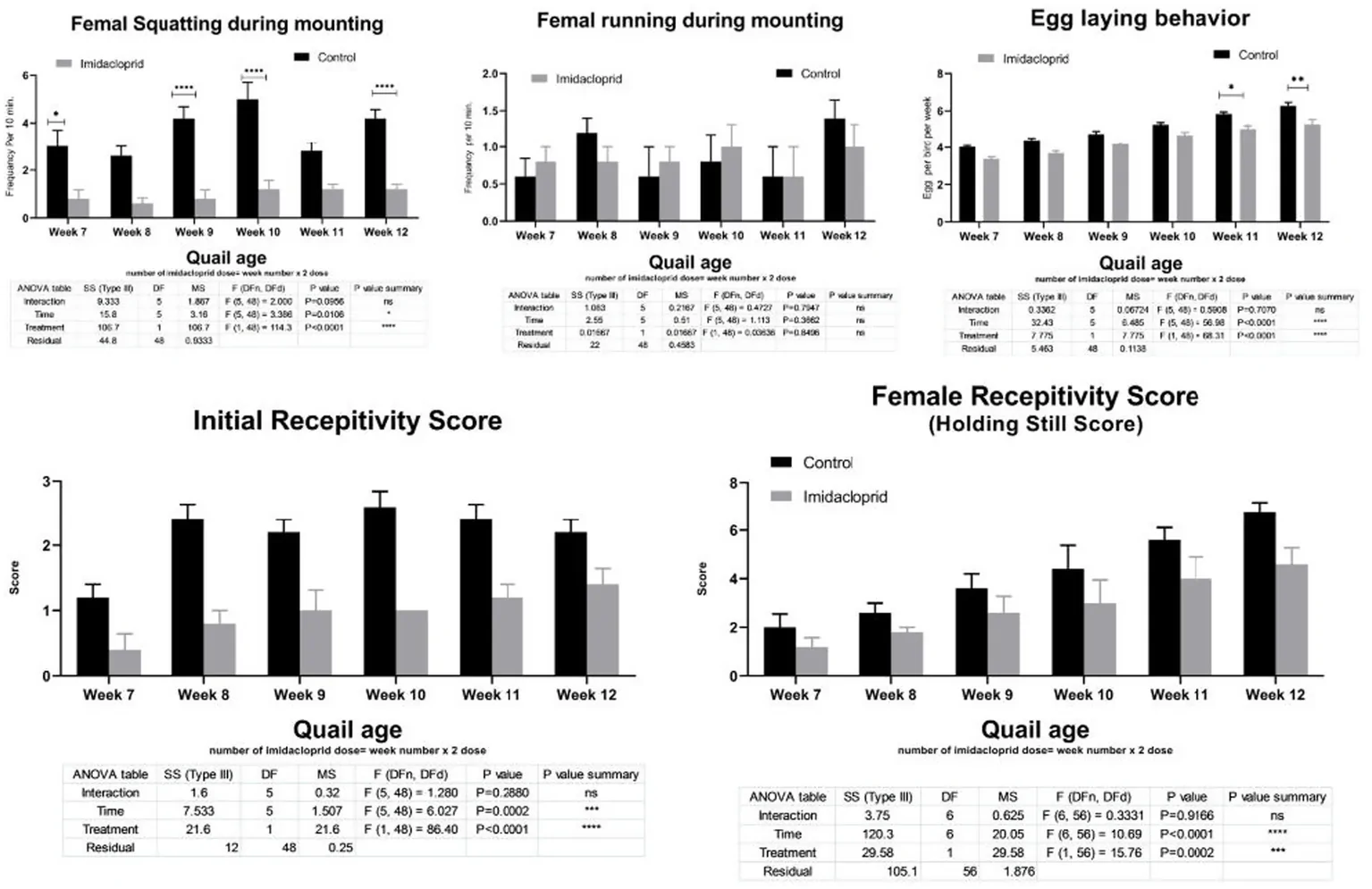
Effects of chronic imidacloprid exposure on sexual receptivity and egg laying performance in female Japanese quail (Coturnix coturnix japonica) across weeks 7 to 12 of age. Data are presented as mean ± SEM. Asterisks denote statistically significant differences between control and imidacloprid-treated groups at individual weeks: *P < 0.05, *P* < 0.01, ***P < 0.001, ****P < 0.0001.

Absolute squatting frequency during mounting attempts was profoundly reduced in treated females, showing significant main effects of treatment [*F*(1, 48) = 114.3, *p* < 0.0001] and time [*F*(5, 48) = 3.386, *p* = 0.0106; interaction: *F*(5, 48) = 2.000, *p* = 0.0956], with marked post-hoc reductions confirmed at Week 7 (*p* < 0.05), Week 9 (*p* < 0.001), Week 10 (*p* < 0.0001), and Week 12 (*p* < 0.0001). In contrast, female running frequency during mounting displays was not significantly altered across treatment [*F*(1, 48) = 0.03636, *p* = 0.8496], time [*F*(5, 48) = 1.113, *p* = 0.3662], or their interaction [*F*(5, 48) = 0.4727, *p* = 0.7947].

Egg laying behavior (eggs per bird per week) exhibited significant main effects of treatment [*F*(1, 48) = 68.31, *p* < 0.0001] and time [*F*(5, 48) = 56.98, *p* < 0.0001; interaction: *F*(5, 48) = 0.5908, *p* = 0.7070], characterized by a progressive age-related rise in laying in control birds that was significantly attenuated in exposed females at Week 11 (*p* < 0.05) and Week 12 (*p* < 0.01). Semi-quantitative receptivity scoring corroborated this inhibition, as the initial receptivity score showed highly significant main effects of treatment [*F*(1, 48) = 86.40, *p* < 0.0001] and time [*F*(5, 48) = 6.027, *p* = 0.0002; interaction: *F*(5, 48) = 1.280, *p* = 0.2880], and the female receptivity score (holding still score) was similarly suppressed by imidacloprid [treatment: *F*(1, 56) = 15.76, *p* = 0.0002; time: *F*(6, 56) = 10.69, *p* < 0.0001; interaction: *F*(6, 56) = 0.3331, *p* = 0.9166], collectively confirming that imidacloprid impairs female receptive posture, passive holding-still compliance, and functional oviposition.

### 5.3 Time-dependent effects of imidacloprid exposure

Across the 6-week experimental period (Weeks 7 to 12 of age), bird age and exposure duration exerted significant main temporal effects on nearly all evaluated parameters in Japanese quail (*Coturnix japonica*). Systemic endocrine profiles showed progressive age-related shifts, with total estrogens, 17beta estradiol, testosterone, progesterone, follicle-stimulating hormone (FSH), and luteinizing hormone (LH) all exhibiting highly significant main effects of time (*p* < 0.0001). In control birds, physiological maturation drove progressive increases in reproductive organ dimensions (cloacal gland area, relative testis weight, and foam production), appetitive courtship activities (waltzing, wing flapping), copulatory behaviors, and female oviposition metrics (egg weight and hen-day egg production) (*p* < 0.001). Conversely, prolonged toxicant exposure interacted significantly with time to alter these developmental trajectories; imidacloprid-treated quails displayed progressive onset of hypo activity, characterized by significantly elevated crouching behaviors starting early (*p* < 0.0001) and systemic sickness behaviors emerging from Week 9 through Week 12 (*p* < 0.001). Furthermore, significant treatment-by-time interactions led to delayed sexual maturity onset, protracted interaction latencies in early maturity (Weeks 7–8), and severe late-stage impairments (Weeks 9–12) in male copulatory efficiency, cloacal contact scores, waltzing frequency, and female weekly egg production (*p* < 0.05 to *p* < 0.0001).

### 5.4 Treatment x time interaction effects

Significant treatment-by-time interactions revealed that imidacloprid exposure fundamentally altered the normal age-dependent trajectory of physiological, reproductive, and behavioral development in Japanese quail (*Coturnix japonica*) across Weeks 7 to 12. Endocrine dynamics were heavily driven by interaction effects, as circulating testosterone [*F*(5, 48) = 5.390, *p* = 0.0005], total estrogens [*F*(5, 48) = 5.000, *p* = 0.0009], and 17beta-estradiol [*F*(5, 48) = 3.302, *p* = 0.0121] showed time-specific suppression patterns, marked by an initial transient rise at Week 7 followed by severe depletion at Weeks 9 through 12. Tissue-to-body mass ratios similarly diverged over time, with female brain-somatic index displaying a highly significant interaction [*F*(5, 48) = 5.557, *p* = 0.0004] characterized by persistent somatic deficits at all observation intervals. Behavioral progression was markedly modified across the exposure window: crouching [*F*(5, 24) = 12.15, *p* < 0.0001], sickness behaviors [*F*(6, 28) = 3.994, *p* = 0.0052], and drinking suppression [*F*(5, 24) = 14.97, *p* < 0.0001] escalated progressively in treated birds, with sickness symptoms becoming particularly prominent from Week 9 onwards. In males, appetitive courtship displays including waltzing [*F*(5, 48) = 3.450, *p* = 0.0096] and wing flapping [*F*(5, 48) = 3.743, *p* = 0.0061] showed marked interaction-driven declines starting at Week 9, while reaction time [*F*(5, 48) = 4.943, *p* = 0.0010] and sexual experience indices [*F*(5, 28) = 3.229, *p* = 0.0200] exhibited phase-specific impairments during early maturity (Week 7) and late-stage performance (Weeks 10–12). Finally, female egg production metrics demonstrated significant interaction effects (*p* ≤ 0.001), as normal age-related gains in egg weight, hen-day egg production, and egg mass were progressively blunted by continuous imidacloprid exposure.

## 6 Discussion

Depressed sexual behavior was the most sensitive endpoint observed in this experiment. Imidacloprid (IMI) exposure in juvenile quails (weeks 1–6 of age) and during maturity (weeks 7–12 of age) resulted in persistent adult sexual and reproductive dysfunction, which was comprehensively evaluated using, genotoxicity, biochemical, reproductive organ-weight, and behavioral parameters.

Assessment of genotoxicity via the alkaline single-cell gel electrophoresis (Comet) assay revealed that subchronic imidacloprid (IMI) exposure induced significant, multi-organ genomic DNA degradation in Japanese quail (*Coturnix coturnix* japonica). In hepatic tissue, IMI administration elicited marked elevations in the percentage of DNA damage (*p* < 0.001), tail length (*p* < 0.05), and tail moment (*p* < 0.001) relative to control birds. Similarly, cerebral tissue from treated quails exhibited a pronounced increase in the percentage of DNA damage (*p* < 0.001) and tail moment (*p* < 0.05). Because tail moment integrates both the physical migration distance (tail length) and the relative proportion of relaxed, broken DNA fragments migrating out of the nucleoid core, it provides a highly dependable and sensitive metric of overall genomic instability and strand-break severity under xenobiotic challenge (45).

The marked genomic damage observed in hepatic tissue directly reflects the role of the liver as the primary anatomical site for xenobiotic biotransformation and detoxification.

During the hepatic metabolism of neonicotinoids, Phase I bioactivation generates substantial levels of reactive oxygen species (ROS) and reactive electrophilic intermediates. When the cellular accumulation of free radicals exceeds endogenous enzymatic and non-enzymatic antioxidant defenses, oxidative stress triggers direct single- and double-strand DNA cleavage, base oxidation, and membrane lipid peroxidation (3, 46). This metabolic bioactivation and subsequent oxidative genotoxicity explain the significant extension in hepatic tail length and tail moment, corroborating previous reports of pesticide-induced genotoxicity and chromosomal fragmentation in avian and mammalian liver systems (47).

Concurrently, the elevated percentage of DNA damage and tail moment in brain tissue confirms that IMI-induced genotoxic stress crosses into the central nervous system, driving significant neuro-genotoxicity (48). The central nervous system is exceptionally vulnerable to oxidative DNA damage due to its high polyunsaturated fatty acid content, elevated rate of oxygen consumption and comparatively lower antioxidant enzyme reserve (49). Chronic overstimulation of nicotinic acetylcholine receptors (nAChRs) by IMI, coupled with acetylcholinesterase (AChE) inhibition, promotes excessive intracellular calcium influx and mitochondrial dysfunction, generating localized ROS cascades that fragment neuronal genomic DNA (46).

This cerebral genomic degradation provides a direct molecular and cellular etiology for the broader structural and functional deficits observed throughout this study:

- Neuroendocrine Dysregulation: Genotoxic injury to neuronal nuclei within the hypothalamus and preoptic area compromises the transcription of essential neurochemical regulators and GnRH synthesis, accelerating the downstream collapse of the HPG axis, gonadotropin release, and gonadal steroidogenesis (12).
- Somatic Organ Hypotrophy: Widespread genomic strand breakage in the brain triggers apoptotic cascades and tissue necrosis, underpinning the severe and persistent depression in the brain-somatic index (BSI) across development (50, 51).
- Behavioral and Motor Collapse: Oxidative genomic damage in higher-order sensorimotor and integrative centers including the forebrain, cerebrum, cerebellum, and septal nuclei directly disrupts the neural circuitry governing voluntary locomotion, courtship displays, and copulatory motor execution (7).

Biochemically, obtained data showed that, chronic exposure to imidacloprid (IMI) precipitated a systemic collapse of circulating gonadotropins and gonadal sex steroids in Japanese quail (*Coturnix coturnix japonica*) across weeks 7 to 12 of age, demonstrating a multi-level disruption of the hypothalamic–pituitary-gonadal (HPG) axis.

In females, obtained data revealed early progesterone depletion at Week 7 (*p* < 0.01) which deprived downstream steroidogenic cascades of essential biosynthetic precursors, contributing to the persistent, unrelenting depletion of total estrogens across Weeks 7, 10, 11, and 12 (*p* < 0.001). Crucially, obtained data of circulating 17β-estradiol exhibited a distinct biphasic response: an initial transient elevation at Week 7 (*p* < 0.01), followed by sharp, sustained suppression from Week 9 onward (*p* < 0.0001). This transient initial surge data may be due to an early compensatory upregulation of ovarian aromatase (*Cyp19a1*) or altered metabolic breakdown via *Cyp2e1* under acute xenobiotic stress (8). However, as chronic exposure progressed, cumulative follicular cytotoxicity, gonadotropin depletion, and precursor exhaustion overwhelmed this adaptive response, driving steroidogenesis into severe exhaustion, which is critical because balanced estrogenic and androgenic signaling is indispensable for vitellogenesis, oviductal functional morphology, and sexually dimorphic neurobehavioral activation (36, 37).

The divergence observed between circulating 17β estradiol levels and total estrogen pools highlights a complex disruption in steroid synthesis and clearance. Rather than representing an experimental contradiction, an elevation in 17β estradiol occurring alongside a decline in total estrogen metabolites may indicate imidacloprid-induced alterations in ovarian cytochrome P450 aromatase (CYP19A1) activity, perturbed conversion of estrone (E1) to (E2) via 17β-hydroxysteroid dehydrogenase (17β HSD), or impaired hepatic hydroxylation and sulfoconjugation clearance pathways (52). Future studies evaluating specific steroidogenic enzyme expression levels are needed to elucidate these precise endocrine dynamics.

Upstream, obtained data showed anterior pituitary secretions were significantly blunted, as evidenced by recurrent depressions in follicle-stimulating hormone (FSH) at Weeks 7, 9, and 10, alongside sustained inhibition of luteinizing hormone (LH) culminating in a marked drop at Week 12 (*p* < 0.01). This gonadotropin suppression data indicates central HPG dysfunction, which may be due to impaired hypothalamic gonadotropin-releasing hormone (GnRH) pulsatility (53) or direct xenobiotic cytotoxicity and apoptotic degeneration within anterior pituitary gonadotropes (54) Moreover,

In male, obtained data demonstrated that circulating testosterone crashed prominently at Weeks 10 (*p* < 0.001) and 11 (*p* < 0.0001). The suppressive effect of IMI on testosterone levels strongly aligns with the previous findings of Mehanna et al. (19), and Mourikes et al. (6).

Mechanistically, this endocrine depression may be driven by IMI-induced disruption of the hypothalamic–pituitary-gonadal axis, which severely compromises the endocrine regulation of both the thyroid and the testes (12).

At the molecular level, IMI has been shown to inhibit testosterone biosynthesis via the marked downregulation of mRNA transcripts for key steroidogenic genes, including *CYP17A1*, *CYP11A1*, *CYP19A1*, *StAR*, and *HSD17B3* (4), alongside a generalized decrease in the transcription of biosynthesis-related testicular genes (20). Furthermore, this drop in hormone synthesis is compounded by localized cytotoxicity within the interstitial tissue. Leydig cells play the primary role in testosterone production and the maintenance of spermatogenesis within the testis and the oxidative loss of interstitial Leydig cells (78). IMI exposure causes severe oxidative damage and extensive degeneration of these cells (55), which drastically impairs their secretory function. This is structurally characterized a marked reduction in Leydig cell density resulting from the disruption of essential cytoskeletal proteins (78). This comprehensive endocrine failure provides the definitive biochemical etiology for the delayed sexual maturation, reduced egg output, and impaired copulatory performance observed in both sexes.

The biochemical data were accompanied by a marked delay in reproductive onset across both sexes. Treated males and females attained sexual maturity significantly later (44.22 vs. 41.90 days in males; 47.44 vs. 45.30 days in females) and at higher mature body weights (149.55 vs. 145.70 g in males; 191.89 vs. 182.90 g in females).

This delay was positively correlated with a decline in both the gonadosomatic index (relative testicular and ovarian weights) and the brain-somatic index. Clinically, this overall increase in the age of sexual maturity manifested as a late onset of egg laying in females and delayed foam production in males. These findings align with previous studies reporting decreased testicular weights (4, 10) and reduced ovarian weights (6), while the delayed puberty under neonicotinoid exposure further supports established avian toxicological reports (56).

In the present study, imidacloprid (IMI)-induced endocrine disruption and organ index changes (GSI and BSI) served as the anatomical basis for delayed sexual maturation. In Japanese quail (*Coturnix coturnix japonica*), pubertal onset is tightly regulated by chronological age alongside critical metabolic and neuroendocrine thresholds (34). Furthermore, functional sexual maturation fundamentally depends on the timely activation of the hypothalamic–pituitary-gonadal (HPG) axis and the surge of circulating gonadotropins and gonadal sex steroids (12).

Physiologically, this developmental arrest may stem from IMI-induced reductions in feed intake, leading to systemic growth retardation and lower body weight (56).

This reduced nutritional intake acts as a form of feed restriction; the subsequent deficit in essential proteins and metabolic energy delays the onset of sexual maturity and triggers a cascade of effects that disrupt standard avian reproductive behaviors.

This developmental delay was structurally underpinned by severe, sexually dimorphic organ hypotrophy, characterized by profound and persistent reductions in relative central and gonadal organ masses across developmental stages (Weeks 7–12). The brain-somatic index (BSI) was significantly depleted in both sexes, exhibiting continuous suppression in females from Week 7 through Week 12 (*p <* 0.0001) and progressive deficits in males that peaked between Weeks 9 and 12 (*p <* 0.0001). Because relative brain mass in birds directly parallels neuronal density, dendritic arborization, and synaptic complexity within higher motor and integrative brain centers (51), this sustained reduction in BSI structurally reflects the extensive neurodegenerative lesions, cerebral necrosis, and genomic DNA degradation documented under chronic neonicotinoid neurotoxicity (49, 50). The heightened vulnerability of females evidenced by immediate, uninterrupted BSI suppression across all evaluated weeks highlights sexually dimorphic neurodevelopmental sensitivity to xenobiotic oxidative stress during pubertal maturation. Concurrently, gonadosomatic indices (GSI) revealed pronounced gonadal regression, characterized by early-onset testicular GSI deficits at Week 7 (*p <* 0.01) in males and prominent ovarian GSI suppression at Week 8 (*p <* 0.001) in females.

Understanding reproductive morphometry is critical, as organ metrics directly dictate reproductive output and species abundance. Testicular size is a powerful predictor of spermatozoa production, fertilization capacity, and sperm storage dynamics (52), serving as a reliable indicator of overall reproductive potential in male Japanese quail (57). Because a strong positive correlation exists between avian testicular growth and overall body weight (58), the IMI-mediated drop in body weight directly suppressed the gonadosomatic index. Given the established connection between testicular volume and pubertal onset, this structural suppression directly caused the delayed sexual maturity observed in treated males. This early testicular atrophy stems from IMI-induced cytotoxicity toward interstitial Leydig cells and the seminiferous epithelium (55), which halts pubertal testicular expansion.

Similarly, the severe suppression of ovarian GSI aligns with xenobiotic-induced follicular atresia, lipid peroxidation within the ovarian stroma, and premature depletion of the primordial follicle reserve (8, 78). Together, these systemic reductions in BSI and GSI illustrate that chronic imidacloprid exposure imposes simultaneous structural damage on central regulatory and peripheral reproductive organs, providing a clear anatomical mechanism for the delayed sexual maturation and impaired copulatory performance observed in both sexes.

Overall sickness behaviors escalated significantly from Week 9 through Week 12 (*p <* 0.001), reflecting cumulative neurotoxicity, oxidative damage, and neuronal necrosis within higher motor and hypothalamic centers (49, 50). This escalation was driven by a profound and unrelenting suppression of drinking behavior across the entire exposure window (*p <* 0.0001), alongside early alterations in feeding patterns and a marked increase in crouching behavior.

The elevation in sickness and crouching behaviors may be attributed to the suppressive effect of imidacloprid (IMI) on plasma testosterone levels (6), as testosterone acts as a crucial modulator of both sickness and active behaviors in male birds (59). The observed increase in crouching across all time points (*p <* 0.0001) serves as a definitive bio-indicator of pesticide-induced hypoactivity, aligning with established reports of IMI-induced crouching in quail models (60). Mechanistically, this hypoactivity stems from neurochemical and structural impairments: persistent agonism of nicotinic acetylcholine receptors (nAChRs) coupled with marked acetylcholinesterase (AChE) inhibition leads to cholinergic overstimulation, receptor desensitization, and neuromuscular junction desynchronization (61). Furthermore, direct axonal degeneration of the sciatic nerve compromises peripheral sensorimotor function (60).

The marked reductions in feeding and drinking align strongly with previous reports by Poliserpi and Brodeur (56), Abd-Elhakim et al. (61), and El-Maguid et al. (60). These behavioral shifts may be driven by reduced testosterone levels, as a direct relationship exists between plasma testosterone concentration and the duration of the free-running period of feeding rhythms in birds (62, 63). Concurrently, elevated 17 beta estradiol levels may further inhibit ingestive behavior, as estradiol exerts potent hypophagic and anti-dipsogenic effects that suppress food and fluid intake (64, 65). Beyond endocrine factors, neonicotinoids are known to disrupt nutrient metabolism and gut health in poultry (66), induce initial ingestion distress (56).

The significant Treatment x time interactions observed for drinking, crouching, and sickness behaviors highlight an age- and dose-dependent aggravation of clinical signs as weekly IMI exposure escalated. This contrasts with studies suggesting acclimation to chronic low-dose exposure, pointing instead to cumulative neural and metabolic stress. Interestingly, while drinking was severely suppressed, feeding behavior exhibited a main treatment effect characterized by elevated bout frequencies in treated quails. Rather than representing true hyperphagia or acute appetite suppression, as commonly reported in short-term songbird exposure models (67), this progressive escalation of sickness behavior from Week 9 through Week 12 directly parallels the findings of Gibbons et al. (68), who demonstrated that chronic environmental exposure to imidacloprid compromises fundamental daily maintenance activities, energy homeostasis, and overall physiological well-being in game birds.

## 7 Male side

Chronic administration of imidacloprid (IMI) severely compromised male reproductive biometrics and functional fertility indices in adult Japanese quail (*Coturnix coturnix* japonica), as demonstrated by significant main effects of treatment, age, and their interaction on cloacal gland area (CAREA), relative testis weight, and foam production (*p* < 0.001 for all). Although these biometric parameters increased progressively from week 7 to week 12 in parallel with normal pubertal development, IMI-treated males consistently exhibited persistent, significant reductions in cloacal gland area (304.66 vs. 332.90 mm), relative testis weight (0.02 vs. 0.03), and secretory foam weight (50.06 vs. 58.50 mg) at every weekly evaluation compared to time matched controls (*p* < 0.001).

In male Japanese quail, testicular size and cloacal gland morphometry serve as non-invasive, reliable bio-indicators of gonadal maturity, functional endocrine status, and overall reproductive capacity (22, 57).

Testicular volume is directly correlated with daily spermatozoa production, seminiferous tubule diameter, and semen storage capacity (52).

The marked, persistent reduction in relative testis weight observed throughout the 6-week observation window due to the obtained decrease testosterone level and testosterone is the primary hormone driving the hypertrophic development of male sexual organs (4) and this reduction reflects direct pesticide induced cytotoxicity and oxidative degeneration within the testicular parenchyma (55), which arrests the standard hypertrophic expansion of seminiferous tubules and interstitial tissue (4).

Concurrently, the marked hypotrophy of the cloacal gland and the sharp decline in foam production are direct anatomical and functional manifestations of systemic androgen deficiency. The cloacal (proctodeal) gland is a strictly androgen dependent secondary sexual structure whose hypertrophic growth and merocrine secretory activity are tightly regulated by local and circulating testosterone concentrations (38, 41). Because cloacal gland surface area is positively correlated with testicular mass and circulating testosterone levels (69), the concurrent drop in testicular weight and steroidogenic output inevitably precipitated glandular atrophy. Consequently, specialized foam output plummeted across all weeks, as foam volume is directly proportional to functional cloacal surface area and glandular secretory epithelial integrity, Given that cloacal gland foam facilitates sperm transport, prolongs sperm viability within the female reproductive tract, and enhances overall fertilization success (41), the sustained suppression of CAREA and foam production provides an anatomical and physiological mechanism for the compromised copulatory efficiency and reduced fertility observed in IMI-exposed males.

## 8 Male sexual behavior

Sexual behaviors in Japanese quail (*Coturnix japonica*) were systematically observed and documented over a six-week period, spanning Weeks 7 through 13 of life. This developmental window was selected because Japanese quail typically achieve sexual maturity between Weeks 7 and 8, reaching fully established baseline reproductive function by Week 10 (34).

The Japanese quail serves as an ideal animal model for investigating testosterone-mediated regulation of sexual behavior (57). Testosterone acts as the primary hormonal driver of this behavioral matrix, both by stimulating the peripheral development of reproductive tissues and by directly modulating the neural nuclei governing sexual drive (7).

In birds, sexual behavior is a complex, highly coordinated process divided into two distinct phases: appetitive behavior and consummatory behavior (36). Appetitive behavior reflects underlying sexual motivation and courtship display, consisting of actions such as waltzing, chasing, and wing flapping. Consummatory behavior encompasses explicit copulatory motor execution and performance, including neck grabs, mount attempts, mounts, and cloacal contact movements.

## 9 Male appetitive courtship and consummatory copulatory behaviors

Subchronic oral administration of imidacloprid (IMI) induced severe, persistent deficits across both the appetitive (courtship) and consummatory (copulatory) phases of male sexual behavior in Japanese quail (*Coturnix coturnix japonica*) from weeks 7 to 12 of age.

Appetitive courtship displays were profoundly blunted, evidenced by persistent reductions in the absolute frequencies of chasing across all weeks (*p* < 0.05 to *p* < 0.0001), alongside sharp declines in waltzing and wing flapping emerging prominently from Week 9 through Week 12 (*p* < 0.01 to *p* < 0.0001). Interestingly, the lack of significant treatment effects on the proportions of chasing, waltzing, and wing flapping relative to total activity reveals that this courtship depression was fundamentally driven by generalized behavioral suppression and overall hypoactivity rather than a selective reallocation of the behavioral budget.

Concurrently, consummatory copulatory performance was consistently inhibited across both home rearing cages and the external testing arena. Exposed males exhibited marked reductions in the absolute frequencies of neck grabs, mount attempts, mounts, and completed cloacal contacts (*p* < 0.01 to *p* < 0.0001). Crucially, proportional behavioral analyses in both environments revealed significant, selective reductions in mounting and cloacal contact proportions highlighted by a pronounced drop in relative cloacal contact efficiency (*p* < 0.0001 in rearing cages; *p* = 0.0340 in the testing arena).

This disparity demonstrates that while IMI suppresses absolute motor output through generalized hypoactivity, it imposes an additional, selective disruption on the neural and motor proficiency required to successfully transition from initial appetitive mounting to terminal cloacal contact (36).

This marked reduction in mounting frequency aligns with the previous findings of Mehanna et al. (19), who reported that IMI significantly prolonged mounting latency and suppressed overall mounting activity in rodent models.

Mechanistically, this consummatory failure may be due to a convergence of neuroendocrine and anatomically obtained data as the severe suppression of circulating testosterone deprives the medial preoptic area (MPOA/POM) and bed nucleus of the stria terminalis (BST) of the essential androgenic activation and local aromatization into 17β-estradiol required to trigger copulatory motor execution (7, 70).

Besides that, the persistent reduction in relative brain weight (BSI) and forebrain neuronal density impairs the acquisition of sexual experience and motor learning (51), rendering exposed males incapable of optimizing their copulatory mechanics over repeated encounters (40). Moreover, previous work data as Histopathological necrosis and genomic DNA damage within the cerebrum, BST, and septal nuclei caused by IMI directly degrade the central coordination of mating sequences (49, 50).

Specifically, lateral and medial septal lesions severely reduce mounting completion while increasing intromission failure and latency (71).

## 10 Male sexual motivation, temporal dynamics, and copulatory competence

Chronic oral administration of imidacloprid (IMI) severely compromised sexual motivation, temporal execution, and overall copulatory competence in male Japanese quail (*Coturnix coturnix japonica*) across Weeks 7 to 12 of age. During initial arena screening encounters, IMI exposure significantly delayed the onset of mating behavior, as evidenced by prolonged latencies to the first sexual interaction at Weeks 7 and 8 (*p <* 0.01) alongside marked increases in reaction time that peaked prominently at Week 10 (*p <* 0.0001). Concurrently, treated males exhibited extended total interaction durations spending longer attempting to engage females without completing copulation and suffered an overall suppression in libido scores (*p =* 0.0003).

This prolonged motivational latency and diminished sexual drive mirror previous findings in rodent and avian models, where neonicotinoids disrupted courtship initiation and prolonged mounting latency (12, 19). These motivational and temporal deficits directly reflect both the depletion of circulating testosterone observed in the present study and the disruption of central dopamine within mesolimbic and hypothalamic regulatory circuits reported in previous studies. Testosterone is essential for stimulating the preoptic area to initiate sexual drive and prime male responsiveness to female conspecific cues (7).

Furthermore, a precopulatory dopamine surge within the medial preoptic area (MPOA) is indispensable for triggering sexual motivation and reducing reaction latencies (72). By depressing both circulating androgens and central dopamine concentrations (49), IMI deprives the brain of the activating neurochemical signals required for prompt sexual arousal and courtship initiation.

At the consummatory level, IMI exposure precipitated a pronounced, persistent failure in functional copulatory execution. Successful mating frequency was severely depressed throughout development, exhibiting marked reductions at Weeks 7, 9, 10, and 12 (*p <* 0.05 to *p <* 0.0001). Crucially, while successful mating collapsed, the frequency of unsuccessful mating attempts showed no significant treatment effect (*p =* 0.0618). This selective outcome demonstrates that IMI neurotoxicity does not simply alter mating kinetics by increasing clumsy or aborted attempts, but fundamentally impairs the neurological competence and motor precision required to achieve successful copulatory completion (cloacal contact). The reduction in successful mating percentages in birds administered IMI aligns with the findings of Angelier et al. (12), who reported that male red munias (*Amandava amandava*) consuming IMI-treated seeds experienced significant impairments in singing, mating, and pairing behaviors during the reproductive season.

Mechanistically, this loss of copulatory efficiency is explained by our findings of reduced relative brain mass (BSI), as compromised forebrain neuronal integrity impairs sensorimotor learning and the execution of acquired sexual experience (51).

## 11 Male sexual dysfunctions

Male sexual dysfunctions represent significant alterations in any phase of the sexual response. Alterations in neural centers, such as hypothalamic lesions or dysfunctions in dopaminergic neurotransmission, are directly associated with disorders of sexual motivation. Similarly, testosterone deficiency reduces the activation of the medial preoptic area (MPOA), thereby compromising the motor execution phase of copulatory behavior (7).

### 11.1 Male sexual proficiency, experience acquisition, and copulatory efficiency

Chronic oral administration of imidacloprid (IMI) severely impaired the developmental acquisition of sexual proficiency, copulatory mechanics, and behavioral efficiency in male Japanese quail (*Coturnix coturnix* japonica) across weeks 7 to 12 of age. The sexual experience index was significantly perturbed by IMI exposure, characterized by abnormally prolonged interaction timelines at Weeks 10 (*p* < 0.05) and 12 (*p* < 0.01). In sexually functional male quail, repeated mating encounters typically drive behavioral optimization: experienced males refine their copulatory motor patterns, initiate courtship rapidly, minimize unproductive contact durations, and achieve terminal cloacal contact with fewer mounts and attempts (40). In contrast, the protracted sexual experience index observed in treated males reflects profound behavioral clumsiness and inefficiency, demonstrating a failure to translate repeated sexual encounters into refined copulatory performance.

This behavioral inefficiency was structurally and functionally corroborated by marked depressions across all semi-quantitative mating scores and consummatory endpoints. Exposed males exhibited persistent reductions in mount scores (*p* < 0.0001), mount attempt scores (*p* < 0.0001), and positive cloacal contact scores (*p* < 0.0001), the latter crashing severely across Weeks 10, 11, and 12 (*p* < 0.001). Correspondingly, absolute cloacal contact movements were significantly depleted by Week 12 (*p* < 0.05). Crucially, copulatory efficiency quantified as the proportion of initiated neck grabs that successfully culminated in completed cloacal contacts was significantly blunted in both home rearing cages (*p* < 0.0001) and the standard testing arena (*p* = 0.0551).

This selective collapse in copulatory completion indicates that while exposed males occasionally initiate appetitive encounters, they possess a severe deficit in executing the terminal motor maneuvers required to achieve functional cloacal apposition (36).

Mechanistically, this loss of sexual proficiency and sensorimotor learning stems from interconnected obtained neurodegenerative and endocrine disruptions:

- Central Neurodegeneration and Forebrain Lesions: The obtained severe depletion of the brain-somatic index (BSI) and the associated loss of forebrain neuronal density directly impair the higher-order sensorimotor learning circuits required for behavioral refinement (49, 51).
- Depleted Androgenic Activation: Systemic testosterone depletion deprives central preoptic circuits (POM/MPOA) of essential activating inputs, blunting neural responsiveness to receptive female cues (4, 7).

Furthermore, the obtained data may be due to agree with previous work as, IMI cause an Axonal neuropathy of the sciatic nerve (60) combined with cholinergic overstimulation via acetylcholinesterase inhibition (61) compromises the physical motor coordination needed to mount and maintain balance. Compounded by marked cloacal gland atrophy and depleted foam production, this neuroendocrine breakdown severely curtails individual fertilization capacity and reproductive fitness. Moreover, IMI cause necrotic damage within the bed nucleus of the stria terminalis (BST) and septal nuclei disrupts the coordination of consummatory execution, elevating intromission latencies and reducing cloacal contact frequency (23, 71).

## 12 Female side

### 12.1 Female reproductive performance, oviposition dynamics, and egg quality

Chronic exposure to imidacloprid (IMI) severely compromised female reproductive output and egg laying dynamics in Japanese quail (*Coturnix coturnix* japonica) across weeks 7 to 12 of age. Two-way ANOVA revealed highly significant main effects of treatment, age, and their interaction (*p* ≤ 0.001 for all) across all evaluated laying parameters, with treated hens exhibiting profound, persistent reductions in total weekly egg numbers (31.7 vs. 36.8), mean egg weight (11.3 vs. 14.7 g), hen-day egg production percentage (HDEP: 62.4 vs. 72.7%), and total egg mass (0.13 vs. 0.40) relative to controls. Although egg weight and HDEP followed physiological developmental trajectories with advancing age, IMI-treated hens consistently produced fewer, lighter eggs and exhibited severely depressed egg mass at every weekly interval (*p* < 0.001). Egg production and egg mass serve as direct functional biomarkers of avian ovarian health, steroidogenic integrity, and systemic metabolic competence (67).

The marked, sustained suppression of oviposition metrics observed throughout the 6-week laying period stems from obtained multi-level toxicological disruptions across the hypothalamic–pituitary-gonadal (HPG) axis and Ingestive Distress and ovarian gonado-somatic index (GSI) atrophy plus previous obtained peripheral reproductive tract:

- Gonadotropin Withdrawal and Follicular Arrest: The systemic downregulation of anterior pituitary follicle-stimulating hormone (FSH) and luteinizing hormone (LH) blunts the cyclical follicular recruitment and maturation hierarchy (53, 54). Lacking adequate gonadotropic stimulation, the avian ovary fails to maintain the continuous pool of pre-ovulatory follicles, directly depressing laying frequency and total egg numbers (18, 78).
- Steroidogenic Disruption and Impaired Vitellogenesis: The profound depletion of circulating progesterone and total estrogens compromises hepatic vitellogenin synthesis and oviductal protein deposition (16, 28). Estrogen is indispensable for mobilizing hepatic yolk precursor lipids and proteins; its chronic exhaustion directly halts adequate yolk accumulation within growing follicles, contributing to the severe reduction in mean egg weight and calculated egg mass.
- Ingestive Distress and Energetic Deficits: Avian egg production imposes immense metabolic and energetic demands. The chronic hypodipsia and anorexic behavioral depression observed in IMI-exposed quail created a negative energy balance that restricted the metabolic resources available for reproductive protein synthesis and shell formation (66, 67).
- Ovarian gonado-somatic index (GSI) atrophy: physically reducing ovarian functional surface area due to neonicotinoid accumulation within ovarian tissue induces severe oxidative stress, lipid peroxidation, and premature apoptosis in developing oocytes and granulosa cells (3, 8). This cellular damage results in the depletion of the primordial follicle reserve and widespread follicular atresia (78).

On the other side, previous obtained data reported that, Imidacloprid-induced tissue degradation and cellular necrosis extend into the magnum and shell gland (uterus) regions (79). Histomorphological alterations within these secretory structures compromise albumen secretion and shell calcification, leading to structurally compromised, lighter eggs (73). Collectively, these findings demonstrate that chronic imidacloprid exposure undermines female avian fecundity through combined neuroendocrine axis suppression, direct ovarian tissue degradation, and disrupted nutrient allocation, culminating in persistent reproductive failure throughout the primary laying cycle.

## 13 Female sexual receptivity, behavioral compliance, and oviposition patterns

The expression of female-typical sexual behavior in Japanese quail is unique; unlike the overt, proactive courtship displays of males, female receptivity is fundamentally characterized by behavioral compliance, passive quiescence, and the willingness to remain stationary during copulatory attempts (36).

In the present study, IMI exposure caused a profound, sustained reduction in absolute squatting frequency across development, marked by significant post-hoc depressions at Weeks 7, 9, 10, and 12 (*p* < 0.05 to *p* < 0.0001). Correspondingly, both initial receptivity scores (*p* < 0.0001) and holding-still compliance scores (*p* = 0.0002) were severely suppressed in treated hens. Crucially, while receptive squatting and passive holding-still compliance collapsed, active evasive behaviors specifically female running frequency during mounting displays showed no significant treatment effect (*p* = 0.8496).

This dissociation demonstrates that IMI does not elicit active avoidance or escape hyperactivity; rather, it selectively abolishes the neuroendocrine mechanisms that drive receptive immobility and posture compliance which agreed with obtained data of total estrogen, 17β-estradiol levels because in avian species, the lordosis-like squatting posture and behavioral quiescence are strictly regulated by ovarian sex steroids, particularly 17β-estradiol and progesterone, which prime hypothalamic and preoptic neural circuits to permit male mounting (37). The chronic suppression of circulating progesterone alongside the long-term exhaustion of total estrogens observed from Week 7 onward deprived central estrogen receptors of essential activating ligands (16). Furthermore, previous research reported that, chronic IMI-induced neurodegeneration and genomic DNA fragmentation within the forebrain and hypothalamic nuclei (48, 49) likely blunted central neural sensitivity to circulating steroids, preventing the execution of the quiescent receptive state essential for successful cloacal contact.

In parallel with behavioral non-compliance, functional oviposition (eggs laid per bird per week) was significantly blunted across the exposure period (*p* < 0.0001). While control hens displayed the normal physiological, age-dependent rise in weekly egg production as maturity advanced, this reproductive trajectory was significantly attenuated in IMI-exposed females, culminating in marked laying deficits at Weeks 11 (*p* < 0.05) and 12 (*p* < 0.01). This progressive reproductive decline aligns with obtained cumulative gonadotropin withdrawal (depressed anterior pituitary FSH and LH) and extensive follicular atresia (53, 78). Lacking adequate gonadotropic support and endogenous estrogens, the ovary fails to maintain the pre-ovulatory follicular hierarchy or synthesize sufficient vitellogenin for yolk deposition (8, 28). Compounded by previously data of histomorphological damage within the magnum and shell gland (79), these neuroendocrine and ovarian insults explain the failure of exposed hens to achieve standard peak laying rates, confirming that chronic imidacloprid exposure simultaneously disrupts both the behavioral compliance required for mating and the physiological machinery governing egg production.

## 14 Time-dependent effects of imidacloprid exposure

The temporal dynamics observed across the 6-week exposure window illustrate how subchronic imidacloprid exposure progressively disrupts the physiological and ethological trajectories of sexual maturation in Japanese quail (*Coturnix japonica*). Under normal developmental conditions, the transition from Week 7 to Week 12 of age marks a critical reproductive window driven by the activation of the hypothalamic–pituitary-gonadal (HPG) axis, as reflected by the age-dependent escalation of circulating gonadotropins (FSH and LH) and sex steroids (74). In control birds, this neuroendocrine surge drove predictable morphometric and behavioral milestones, including expansion of androgen-dependent structures (cloacal gland area and foam production), heightened appetitive courtship displays, and optimized oviposition performance (75). Conversely, continuous imidacloprid administration severely blunted these physiological gains through both main temporal effects and treatment-by-time interactions (68).

The progressive emergence of sickness behaviors and crouching from Week 9 onward highlights a cumulative toxic burden, suggesting that prolonged neonicotinoid exposure exceeds physiological clearance capacities and induces systemic neurotoxicity (67). The early onset of prolonged mating latencies (Weeks 7–8) points to an initial impairment in appetitive motivation and sensory processing, whereas the severe late-stage deterioration in copulatory efficiency, cloacal contact scores, and egg mass (Weeks 9–12) reflects structural and functional breakdown of gonadal tissue and central neuroendocrine signaling pathways (76). By blunting normal maturation curves, subchronic imidacloprid exposure not only induces immediate reproductive and behavioral deficits but also fundamentally alters the developmental trajectory required for successful reproductive fitness.

## 15 Treatment x time interaction effects

The significant treatment-by-time interaction effects demonstrate that continuous subchronic exposure to imidacloprid does not merely induce static baseline deficits, but actively destabilizes the chronological maturation profile of Japanese quail (*Coturnix japonica*). In control populations, the period from Week 7 to Week 12 corresponds to peak hypothalamic–pituitary-gonadal (HPG) axis activation, characterized by progressive endocrine maturation, structural gonadal enlargement, and the refinement of complex motor routines essential for courtship and oviposition (74). Imidacloprid exposure fundamentally altered these developmental arcs. The initial transient elevation of 17beta-estradiol at Week 7 followed by severe late-stage depletion of circulating sex steroids (testosterone and total estrogens) at Weeks 9–12 suggests a biphasic neuroendocrine disruptional profile likely reflecting early homeostatic compensation or acute transient enzyme upregulation, followed by progressive functional exhaustion of central gonadotropin signaling and gonadal steroidogenic machinery (76).

This time-dependent neuroendocrine collapse directly translated into a progressive behavioral and somatic decline. The interaction-driven divergence in female brain-somatic index across all observation intervals highlights persistent neurodevelopmental and organ-specific mass deficits relative to total body weight, pointing to systemic metabolic stress and microstructural central nervous system damage (68). Ethologically, the cumulative toxic burden became overwhelmingly pronounced from Week 9 onwards, marked by escalating crouching, reduced drinking, and generalized sickness behavior. This temporal shift tightly correlated with the sharp declines observed in male appetitive courtship displays (waltzing and wing flapping) and copulatory performance (reaction time and sexual experience index), as well as the complete blunting of normal age-related gains in female egg weight, hen-day egg production, and total egg mass (67, 75). Taken together, these interaction dynamics confirm that imidacloprid toxicity accumulates over time, transforming subtle early-stage developmental disruptions into systemic reproductive, behavioral, and neuroendocrine collapse as birds reach full physical.

## 16 Study limitations and future perspectives

While this study provides comprehensive insights into the multi-systemic toxicity of imidacloprid in Japanese quail, several limitations should be acknowledged. First, this research was conducted under controlled laboratory conditions with optimized husbandry and a fixed exposure regimen. In natural settings, wild avian species encounter fluctuating environmental stressors, dietary variability, and seasonal shifts that can significantly alter pesticide toxicokinetics and behavioral outcomes. Second, our external arena behavioral assessments relied on brief observation windows, which may not fully capture subtle, long-term shifts in social dynamics and mating hierarchies over an extended breeding season. Although observing behavior within home rearing cages partially mitigated this limitation, continuous or extended monitoring periods are needed to capture comprehensive behavioral profiles. Finally, while severe disruptions in egg production and reproductive morphometric were established, this study did not evaluate egg hatchability or potential transgenerational impacts on offspring survival and fitness. Future longitudinal studies are warranted to assess the transgenerational consequences of chronic exposure and determine whether the observed genotoxicity and endocrine disruption persist following a depuration period.

## 17 Conclusion

In conclusion, this study demonstrates that imidacloprid exposure exerts severe, multi-systemic toxicity in both male and female quails, significantly compromising their developmental, reproductive, and genetic integrity. The pesticide acts as a potent endocrine disruptor, severely altering sex steroid dynamics and gonadotropins, which clinically manifests as marked gonadosomatic degradation and diminished productive performance in females. Crucially, these physiological impairments are mirrored by profound behavioral shifts, including a surge in debilitating sickness behavior and a stark decline in courtship, copulatory efficiency, and sexual receptivity.

Furthermore, the significant increase in single-cell DNA fragmentation within the liver and brain highlights the severe genotoxic threat imidacloprid poses to avian species. By linking structural and genetic biomarkers directly with functional behavioral modifications, these findings underscore the profound ecological risk that neonicotinoid insecticides pose to wild avian populations in agricultural landscapes. Ultimately, this research emphasizes the critical necessity of integrating behavioral ecotoxicology into multi-tier regulatory risk assessments to safeguard avian biodiversity and ecosystem health.

## Data Availability

The datasets used during the current study are available from the corresponding author on reasonable request.
