# Supplementary material for: Sexual behavior dysfunction and sickness behavior as an early warning indicators of imidacloprid exposure, associated with pituitary–adrenal axis dysregulation and DNA damage in Japanese quails
Source: Front Vet Sci. 2026 Oct 16;13:1914494. doi: 10.3389/fvets.2026.1914494 (PMC13756597; doi:10.3389/fvets.2026.1914494)
Supplement: Supplementary file 1 [file Supplementary_file_1.doc]

Figure 1 show diet table
