# Supplementary material for: Sexual behavior dysfunction and sickness behavior as an early warning indicators of imidacloprid exposure, associated with pituitary–adrenal axis dysregulation and DNA damage in Japanese quails
Source: Front Vet Sci. 2026 Oct 16;13:1914494. doi: 10.3389/fvets.2026.1914494 (PMC13756597; doi:10.3389/fvets.2026.1914494)
Supplement: Supplementary file 4 [file Supplementary_file_4.doc]

**Figure 4. Representative photomicrographs of single-cell gel electrophoresis (alkaline comet assay) demonstrating genomic DNA damage in brain tissue of Japanese quail (*Coturnix japonica*) following 12 weeks of imidacloprid administration.** Fluorescent comet images stained with ethidium bromide show intact, spherical nucleoids with minimal DNA migration in control birds (top left), whereas Japanese quail exposed to imidacloprid (4.43 mg/kg, twice weekly for 12 weeks) exhibit marked DNA strand breakage characterized by distinct comet tail formation, elevated tail DNA percentage, and increased tail moment across representative micro-fields (Scale bar = 50 µm).

**Figure 5. Representative photomicrographs of single-cell gel electrophoresis (alkaline comet assay) demonstrating hepatic genomic DNA damage in Japanese quail (*Coturnix japonica*) following 12 weeks of exposure to imidacloprid.** Ethidium bromide-stained fluorescent comet images of control liver tissue (top row) show intact, round nucleoids with smooth margins and non-migrated DNA, whereas liver tissue from Japanese quail treated with imidacloprid (IMI Liver; bottom row, 4.43 mg/kg twice weekly for 12 weeks) displays substantial hepatic DNA fragmentation, characterized by prominent comet tail migration, increased tail DNA percentage, and visual nuclear dispersion relative to the control group (Scale bar = 50 µm).
