# Supplementary material for: Sexual behavior dysfunction and sickness behavior as an early warning indicators of imidacloprid exposure, associated with pituitary–adrenal axis dysregulation and DNA damage in Japanese quails
Source: Front Vet Sci. 2026 Oct 16;13:1914494. doi: 10.3389/fvets.2026.1914494 (PMC13756597; doi:10.3389/fvets.2026.1914494)
Supplement: Supplementary file 5 [file Table_1.doc]

**Supplementary Material**

**Supplementary Table**

*Table I. Ethogram of sickness and active maintenance behaviors in Japanese quail*

| **Behavioral Domain** | **Behavior / Variable** | **Operational Definition (Physical Criteria & Posture)** | **Quantification & Sampling Unit** | **Reference Adaptation** |
| --- | --- | --- | --- | --- |
| General / Sickness | Crouching (Sickness Behavior) | Remaining in a continuous, stationary recumbent posture for >3 s with the ventral abdomen positioned horizontally in direct physical contact with the cage substrate. | Cumulative Frequency & Duration (s) | Adapted from Gormally et al. (2022) |
|  | Eating Bout | Directing beak pecks into the feed trough; initiated at first peck into the feeder and terminated upon active physical displacement of the head/body away from the feeder area. | Discrete Bouts (Frequency & Duration) | Standard ethological criteria |
|  | Drinking Bout | Directing beak pecks into the water cup; initiated at first contact with water source and terminated upon active physical displacement away from the water receptacle. | Discrete Bouts (Frequency & Duration) | Standard ethological criteria |
|  |  |  |  |  |
