# Supplementary material for: Sexual behavior dysfunction and sickness behavior as an early warning indicators of imidacloprid exposure, associated with pituitary–adrenal axis dysregulation and DNA damage in Japanese quails
Source: Front Vet Sci. 2026 Oct 16;13:1914494. doi: 10.3389/fvets.2026.1914494 (PMC13756597; doi:10.3389/fvets.2026.1914494)
Supplement: Supplementary file 6 [file Table_2.doc]

***Table 2. Ethogram of male sexual behaviors in Japanese quail***

| **Behavioral Domain** | **Behavior / Variable** | **Operational Definition (Physical Criteria & Posture)** | **Quantification & Sampling Unit** | **Reference Adaptation** |
| --- | --- | --- | --- | --- |
| Male Sexual: Appetitive (Courtship) | Waltzing | Locomotion in a circular path around the female while extending and lowering the wing contralateral to her position toward the ground. | Frequency (Count) | Hafez et al. (2023); Suzuki & Tsukahara (2025) |
|  | Wing Flapping | Rapid vertical elevation and rhythmic oscillation of both wings above the dorsal midline while remaining stationary or walking. | Frequency (Count) | Hafez et al. (2023); Suzuki & Tsukahara (2025) |
|  | Chasing | Direct forward locomotion following immediately behind a female for ≥2 consecutive spatial steps. | Frequency (Count) | Hafez et al. (2023); Suzuki & Tsukahara (2025) |
| Male Sexual: Consummatory (Copulatory) | Neck Grab | Approaching the female from behind and firmly clasping her nuchal/cervical feathers between upper and lower mandibles. | Frequency (Count) | Hafez et al. (2023); Palacios-Arellano et al. (2025) |
|  | Mounting | Climbing onto the female’s dorsum and securing foot placement on her wing bases or back feathers. | Frequency (Count) | Suzuki & Tsukahara (2025) |
|  | Mount Attempt | Execution of a neck grab combined with an immediate attempt to climb onto the female's dorsum. | Frequency (Count) | Gormally et al. (2022) |
|  | Full Copulation (Cloacal Contact) | Dorsal positioning on the female accompanied by rhythmic pelvic treading movements, culminating in complete apposition of male and female vent regions. | Frequency (Count) | Suzuki & Tsukahara (2025) |
|  |  |  |  |  |
