# Supplementary material for: Sexual behavior dysfunction and sickness behavior as an early warning indicators of imidacloprid exposure, associated with pituitary–adrenal axis dysregulation and DNA damage in Japanese quails
Source: Front Vet Sci. 2026 Oct 16;13:1914494. doi: 10.3389/fvets.2026.1914494 (PMC13756597; doi:10.3389/fvets.2026.1914494)
Supplement: Supplementary file 7 [file Table_3.doc]

**Table 3. Ethogram of male sexual performance in Japanese quail**

| **Behavioral Domain** | **Behavior / Variable** | **Operational Definition (Physical Criteria & Posture)** | **Quantification & Sampling Unit** | **Reference Adaptation** |
| --- | --- | --- | --- | --- |
| Temporal & Kinematic Parameters | Latency to First Sexual Interaction | Elapsed time between the introduction of the male into the test arena and the exact moment of the first neck grab. | Time / Duration (s) | Pu et al. (2022) |
|  | Reaction Time | Elapsed time between male introduction into the test arena and successful mounting onto the female's dorsum. | Time / Duration (s) | Akinola et al. (2025) |
|  | Sexual Interaction Duration | Continuous time elapsed from the initiation of the first neck grab until either a mount attempt or full cloacal contact is completed. | Time / Duration (s) | Pu et al. (2022) |
|  | Overall Interaction Duration | Cumulative time spent in close physical proximity (<5 cm) to the female, including active chasing phases with or without tactile contact. | Time / Duration (s) | Extended temporal parameter |
| Libido & Cohort Indices | Libido Score | Total number of completed mount attempts executed by a single male within the initial 2-min screening window. | Score / Frequency (Count) | Akinola et al. (2025) |
|  | Sexual Performance Index | Number of positive male responders exhibiting sexual behaviors per treatment group cohort, evaluated on a categorical scale from 0 to 5. | Categorical Index (0–5) & Cohort % | Pu et al. (2022) |
