# Supplementary material for: Sexual behavior dysfunction and sickness behavior as an early warning indicators of imidacloprid exposure, associated with pituitary–adrenal axis dysregulation and DNA damage in Japanese quails
Source: Front Vet Sci. 2026 Oct 16;13:1914494. doi: 10.3389/fvets.2026.1914494 (PMC13756597; doi:10.3389/fvets.2026.1914494)
Supplement: Supplementary file 8 [file Table_4.doc]

Table 4. Ethogram of female sexual behaviors in and receptivity scoring Japanese quail

| Behavioral Category | Parameter | Definition / Description | Unit / Measurement Scale | Reference |
| --- | --- | --- | --- | --- |
| Female Receptive Responses | Receptive Squatting | Lowering the body posture into a stationary, dorso-ventrally flattened position or remaining completely motionless beneath the male during a copulatory attempt. | Frequency (Count) | Suzuki & Tsukahara (2025) |
|  | Non-Receptive Avoidance | Active acceleration or lateral displacement away from an approaching male during a mounting or neck grab attempt to prevent physical contact. | Frequency (Count) | Suzuki & Tsukahara (2025) |
| Female Receptivity Scales | Immobility (Holding Still) Score | Assessment of physical compliance based on stationary time proportion:• Score 0: Highly active; moved constantly.• Score 1: Low immobility (<50% stationary).• Score 2: Moderate immobility (>50% stationary).• Score 3: Complete immobility (100% stationary throughout). | Scale (0–3) | Marin and Satterlee (2003) |
|  | Initial Receptivity Score | Immediate behavioral reactivity following male introduction:• Score 0: Highly unreceptive; constantly ran away and refused to squat.• Score 1: Unreceptive; avoided male, but not markedly.• Score 2: Ambiguously receptive; intermediate/mixed responses.• Score 3: Clearly receptive; actively squatted and remained stationary. | Scale (0–3) | Rutkowska and Adkins-Regan (2009) |
